# An Innovative Blend of Cinnamon Essential Oil, Roselle, and Moringa Flower Powders as a Natural Alternative to Sodium Nitrite in Chicken Sausage Processing

**DOI:** 10.3390/foods15193450

**Published:** 2026-09-27

**Authors:** Annada Das, Subhasish Biswas, Kaushik Satyaprakash, Dipanwita Bhattacharya, Pramod K. Nanda, Gopal Patra, Arun K. Bhunia, Arun K. Das

**Affiliations:** 1 Department of Livestock Products Technology, Faculty of Veterinary and Animal Sciences, West Bengal University of Animal and Fishery Sciences, 37 Belgachia Road, Kolkata 700037, West Bengal, India; dasannada.555@gmail.com (A.D.); lptsubhasish@gmail.com (S.B.); lptgopal81@gmail.com (G.P.); 2 Institute of Veterinary Science and Animal Husbandry, Campus 4, Siksha ‘O’ Anusandhan University, Bhubaneswar 751029, Odisha, India; 3 Faculty of Veterinary and Animal Sciences, Banaras Hindu University, Rajiv Gandhi South Campus, Mirzapur 231001, Uttar Pradesh, India; kaushik_vph@bhu.ac.in (K.S.); dr.dipanwita.vet@gmail.com (D.B.); 4 Eastern Regional Station, Indian Council of Agricultural Research-Indian Veterinary Research Institute, 37 Belgachia Road, Kolkata 700037, West Bengal, India; npk700@gmail.com; 5 Molecular Food Microbiology Laboratory, Department of Food Science, Purdue University, West Lafayette, IN 47907, USA; 6 Purdue Institute of Inflammation, Immunology and Infectious Disease, Purdue University, West Lafayette, IN 47907, USA; 7 Department of Comparative Pathobiology, College of Veterinary Medicine, Purdue University, West Lafayette, IN 47907, USA

**Keywords:** nitrite alternative, cinnamon essential oil, moringa flower, roselle flower, chicken sausages, food safety, shelf life, sensory

## Abstract

The use of nitrite and nitrate in processed meats is a public health concern because they can form potentially carcinogenic N-nitroso compounds. This study developed and characterized a natural phytochemical formulation comprising roselle flower and moringa flower powder, and cinnamon essential oil (5.5:4:0.5) as a potential nitrite alternative in emulsion-based chicken sausages. The formulation exhibited strong antioxidant activity, with high total phenolic content (147.44 mg GAE/g) and effective DPPH radical-scavenging capacity. Gas chromatography–mass spectrometry (GC–MS) analysis preliminarily identified diverse bioactive secondary metabolites. It also demonstrated broad-spectrum antimicrobial activity against major foodborne pathogens and remained stable across varying pH (2–6) and temperature (37–100 °C) conditions. Toxicity evaluations, including red blood cell hemolysis, probiotic compatibility, and Vero cell cytotoxicity assays, confirmed its non-toxic nature. Chicken sausages containing different levels of the phytochemical formulation (0–1%) and sodium nitrite (0–150 ppm) were stored at 4 ± 1 °C for 20 days and evaluated periodically. Compared with controls, sausages containing the formulation showed significantly (*p* ≤ 0.05) improved water retention (22% vs. 25%), nutritional quality (moisture = 66.02 vs. 67.24%, total ash = 2.09 vs. 2.32%, and carbohydrate = 2.76 vs. 2.98%), and texture (1.88 vs. 1.80 kgf/cm^2^ on day 0 and 1.44 vs. 1.64 kgf/cm^2^ on day 20), while exhibiting lower lipid oxidation (0.287 vs. 0.278 mg MDA/kg on day 0 and 2.031 vs. 0.761 mg MDA/kg on day 20), residual nitrite levels (80.35 vs. 31.10 mg/kg on day 0 and 46.96 vs. 16.03 mg/kg on day 20), and microbial counts [total viable count (1.58 vs. 1.28 log CFU/g on day 0 and 4.65 vs. 3.52 log CFU/g on day 20), psychrophiles (3.56 vs. 1.24 log CFU/g on day 20), coliforms (undetectable), *Staphylococcus aureus* (2.55 vs. 1.34 log CFU/g on day 20)]. Although complete nitrite replacement produced an undesirably dark color, partial replacement (1/2 to 2/3, i.e., 75–100 ppm nitrite) maintained product quality, and shelf life, highlighting the formulation’s potential for cleaner-label, healthier processed meat products.

## 1. Introduction

Emulsion-based cooked chicken sausages are widely consumed and have become an important part of modern diets because consumers prefer leaner meat, convenience, affordability, and desirable sensory attributes. However, their quality, safety, and acceptability depend heavily on chemical preservatives, especially sodium nitrite, which is valued for its multifunctional properties. This additive imparts the characteristic pink color by forming the cured pigment nitrosohemochrome and inhibits lipid oxidation by preventing rancidity and “warmed-over flavor” in processed meat [1]. Further, it enhances flavor and suppresses the growth of pathogenic microorganisms, particularly *Clostridium botulinum* [2]. About 100–200 ppm of nitrite can sufficiently inhibit *Clostridium* spp., *Bacillus* spp., *Listeria monocytogenes*, *Salmonella enterica* serovar Typhimurium, *Escherichia coli*, *Staphylococcus aureus*, and others. In contrast, up to 300 ppm is required to inhibit *C. botulinum* toxin production in processed meat products [3].

Despite its beneficial functions, nitrite in processed meat has often been linked to adverse effects on human health, such as esophageal, nasopharyngeal, gastric, colorectal, brain, and bladder cancers [4]. In addition, it can cause methemoglobinemia or “blue baby syndrome” in infants [2]. Further, the interaction of nitrite and secondary amines is responsible for the formation of carcinogenic N-nitroso compounds (e.g., nitrosamines) during different stages in the processing and storage of meat products [5,6]. The International Agency for Research on Cancer (IARC) under the World Health Organization (WHO) has classified processed meat as carcinogenic (Group 1) [7]. Excessive nitrite intake causes enlargement of the thyroid gland and may also interfere with progesterone synthesis, contributing to abortion [8]. In response, regulatory authorities have imposed strict limits on allowable nitrite levels in meat products. The maximum permissible limit (MPL) of nitrite in processed meat formulations is 150 ppm, as per the European Union (EU) [9]. Further, consumer demand for “clean-label” foods free of synthetic additives has increased, as a recent survey suggests that consumers now seek “nitrite-free” labels on meat products, due to increased focus on health and wellness [10].

As efforts to replace or reduce synthetic nitrites with clean-label strategies continue, natural ingredients emerge as the most promising nitrite alternatives, aligning with “clean label” demands because they are free of E-numbers [11,12]. Further, most plant-based ingredients exhibit nitrite-mimicking properties, have a long history of use, and have gained GRAS (Generally Recognized As Safe) status from the Food and Drug Administration (FDA) [7].

Over the years, researchers have explored several plant-derived ingredients and essential oils (EOs), with excellent antioxidant, antimicrobial, and color-enhancing properties, as natural nitrite alternatives in processed meat products. For example, a mixture of fruit and vegetable powders containing Chinese cabbage, celery, and cranberries was demonstrated to improve the physicochemical quality of fried beef meatballs [13]. Pitaya peel (PP) extract and lemon seed essential oil (LEO) could also partially replace nitrite without affecting the physicochemical, microbial, and sensory properties in cured mutton [2]. Ozaki et al. [14] used radish and beetroot powders (0.5% and 1.0%, respectively) as nitrite substitutes in fermented cooked sausages. Coriander EO (CEO) (0.075–0.150 µL/g) with different levels of sodium nitrite (0, 50 and 100 mg/kg) was demonstrated to be effective in cooked pork sausages [6]. Efforts have also focused on nitrite-rich vegetable powders such as celery or beetroot, combined with starter cultures to serve as chemical nitrite substitutes and generate nitrite in situ in cured meat products. However, these alternatives cannot fully eliminate nitrite-related concerns, as residual nitrite persists and continues to contribute to the formation of N-nitroso compounds [11]. Therefore, finding the desirable combination and quantity of plant extracts and EOs with synergistic effects is important, as a single plant ingredient cannot replicate the multifunctionality of synthetic nitrite, offering antimicrobial, antioxidant, and color-stabilizing properties in processed meat products.

In our previous study [15], we developed a novel phytochemical combination from three plant ingredients: roselle (*Hibiscus sabdariffa*) flower powder (RFP), moringa (*Moringa oleifera*) flower powder (MFP), and cinnamon (*Cinnamomum verum*) essential oil (CmEO). Antioxidant activity was estimated using the 2,2-azino-bis (3-ethylbenzothiazoline-6-sulfonic acid) (ABTS.^+^) radical cation decolorization assay and the Ferric reducing antioxidant power (FRAP) assay. The different functional groups present in the phytochemical combinations were identified using Fourier Transform Infrared (FTIR) Spectroscopy. The efficacy of this phytochemical blend in chicken sausages was preliminarily evaluated during chilled storage mainly through some physicochemical quality parameters (pH, water activity, peroxide value, and total volatile basic nitrogen), instrumental color values, and structural integrity (Scanning Electron Microscope -SEM) and light microscope by hematoxylin and eosin (H&E) staining) [15].

However, the study [15] did not address detailed properties of the chosen formulation, including antimicrobial activity, toxicity assessment, and its effectiveness in reducing residual nitrite while maintaining nutritional, textural, microbiological, and sensory qualities. Therefore, in the current study, we conducted a comprehensive in vitro characterization of the previously developed phytochemical formulation comprising roselle flower and moringa flower powders and cinnamon essential oil (5.5:4:0.5) using Total Extractable Components (TEC), pH, color, and residual nitrite (RN) content. GC–MS was used to preliminarily identify bioactive secondary metabolites in the phytochemical formulation. Antioxidant assays based on total phenolic content and 2,2-Diphenyl-1-picrylhydrazyl (DPPH) radical scavenging assay and antimicrobial assays against some common Gram-positive and Gram-negative foodborne bacteria, including anti-clostridial (surrogate) activity, minimum inhibitory concentration (MIC), minimum bactericidal concentration (MBC), DNA degradation analysis, time-kill, and stability assays, were evaluated. We also performed toxicity assessments, including hemolysis, probiotic compatibility, and Vero cell cytotoxicity assays. We further evaluated the formulation’s functional efficacy in a meat model system (chicken sausages) during refrigerated storage (4 ± 1 °C) for 20 days to assess its potential as a natural nitrite alternative in processed meat products. The sausages were evaluated for expressible water, proximate composition, and calorific value on day 0 of sausage processing. Various physicochemical quality parameters, including Thiobarbituric Acid Reacting Substances (TBARS) values, residual nitrite (RN) content, shear force value, total pigment, cured pigment (nitrosyl haemochrome), curing efficiency (%), microbiological quality indicators (Total Viable Count, psychrophilic count, coliform count, and *S. aureus* count), and sensory quality attributes (color and appearance, flavor, texture and tenderness, juiciness, overall acceptability) were estimated during storage. Our results show that partial nitrite replacement (75–100 ppm) preserved product quality, safety, and shelf life, demonstrating its potential for cleaner-label, healthier processed meat products.

## 2. Materials and Methods

### 2.1. Preparation of Phytochemical Formulation

Fresh plant parts used in this study were obtained from local markets (Kolkata, India) and identified by a botanist, an ex-professor at Samanta Chandra Sekhar (Autonomous) College, Puri, affiliated with Utkal University, Odisha, India. Cinnamon essential oil was purchased from RV Essential (New Delhi, India). The individual plant powders and their ethanolic extracts (70% *v*/*v*) were prepared as before [16].

Three different formulations were prepared by blending roselle flower powder (RFP), moringa flower powder (MFP), and cinnamon essential oil (CmEO) at different ratios: (4:4:2), (5.5:4:0.5), and (5:4:1) (% *w*/*w*). Preliminary studies on stability, color development, antioxidant capacity, and antimicrobial efficacy showed that the formulation ratio (5.5:4:0.5) was the most effective and this ratio was therefore used in subsequent experiments.

For in vitro characterization studies, 70% (*v*/*v*) ethanolic extract of the chosen blend/combination (5.5:4:0.5), designated as “formulation,” was used as described [15], whereas its powder form (% *w*/*w*) was used directly in chicken sausage for product development studies.

### 2.2. Characterization of Phytochemical Formulation

#### 2.2.1. Total Extractable Components (TEC), pH, Color, and Residual Nitrite (RN) Content

The formulation was analyzed for Total Extractable Components (TEC %), pH, instrumental color, and residual nitrite (RN) content. TEC % or extraction yield (%) was calculated [17] using the formula

$$TEC\;\left(\%\right)\;or\;extraction\;yield\left(\%\right)=\frac{Final\;weight\;of\;extracted\;sample}{weight\;of\;initial\;sample}\times 100$$

The pH was checked using a digital pH meter (pH tutor, Eutech Instruments, Singapore). The instrumental color coordinates [lightness (L*), redness (a*), yellowness (b*), hue angle (h_ab_), and chroma (C*_ab_)] were measured using a ColorQuest XE colorimeter (HunterLab, Reston, VA, USA). The RN was determined by the method of AOAC [18] and calculated by the following formula:

$$NaNO_{2}\;content\;(mg/kg)=\frac{C\times 2000}{M\times V}$$

where C = concentration of NaNO_2_ (ppm) from the calibration curve that corresponds to the absorbance of the sample; M = mass (g) of sample; V = volume (mL) of sample filtrate.

#### 2.2.2. Total Phenolics Content (TPC) and 2, 2-Diphenyl-1-Picrylhydrazyl (DPPH) Assay

The TPC was estimated by the classical Folin–Ciocalteu (FC) method [19] and was expressed as mg/g of gallic acid equivalent (GAE). The DPPH radical-scavenging activity (RSA%) was assayed using the method of Brand-Williams et al. [20] using the following formula:

$$DPPH\;RSA\;(\%)=\frac{A_{B}-A_{S}}{A_{B}}\times 100$$

where A_B_ = absorbance of the blank and A_S_ = absorbance of the sample. The half-maximal inhibitory concentration (IC_50_) was determined by plotting RSA (%) against the sample concentrations.

#### 2.2.3. Gas Chromatography–Mass Spectrometry (GC–MS) Analysis

Preliminary identification of compounds in the phytochemical formulation was done using a gas chromatograph–mass spectrometer (Shimadzu GCMSQP2010, Nakagyoku, Kyoto, Japan; runtime was ~70 min) and GC–MS solution software (Version 4.52, Shimadzu Sci, Nakagyoku, Kyoto, Japan) [21], and the identity was determined by matching its mass spectra to standards from the NIST library (Version 20, Gaithersburg, MD, USA).

#### 2.2.4. Agar Well Diffusion Assay, MIC, MBC and Tolerance Level

The formulation was evaluated for antimicrobial activity by an agar well diffusion assay against *S. aureus* ATCC 25923, *L. monocytogenes* ATCC 19111, *L. monocytogenes* ATCC 13932, *S. typhimurium* ATCC 14028, *E. coli* ATCC 25922, *C. perfringens* MTCC 450 and *C. sporogenes* MTCC 2684, as per the method of Perez et al. [22] and the Clinical and Laboratory Standards Institute (CLSI) guidelines [23].

The MIC and MBC of the formulation were determined against some Gram-positive and Gram-negative bacteria. The MIC was determined by the resazurin dye reduction method. The MBC was determined by plating on Mueller–Hinton agar (MHA) plates by the drop plate method [24]. The mechanism of antibiosis (bactericidal or bacteriostatic) was ascertained by calculating the MBC/MIC ratio, or tolerance level [25].

#### 2.2.5. Bacterial DNA Degradation, Dose- and Time-Dependent In Vitro Growth Kinetics and Stability Assays

The extent of damage caused by the formulation to genomic and plasmid DNA (pUC19) was assessed by agarose gel electrophoresis, as described previously [26]. The in vitro growth kinetics of the *S. aureus* (SA) ATCC 25923 isolate were evaluated by time-kill assay using the formulation (at 1X MIC and 1X MBC), with the log-phase bacterial culture (inoculum adjusted to 1 X 10^7^ CFU/mL) incubated in cation-adjusted Mueller–Hinton broth (CAMHB). A 50 µL inoculum was transferred to the tubes containing 1X MIC and 1X MBC of the phytochemical formulation, and the tubes were incubated at 37 °C. The isolate treated with ciprofloxacin (1X MIC and 1X MBC) served as the treatment control, while the target bacterial isolate in CAMHB served as the untreated control. Aliquots of 10 µL from each were collected at fixed time intervals (0, 3, 6, 9, 12, and 24 h), diluted 10-fold in sterile phosphate-buffered saline (PBS), and plated on MHA plates [24]. The inoculated agar plates were incubated at 37 °C for 24 h, and the colony counts were expressed as log_10_ CFU/mL. To assess the dose- and time-dependent in vitro growth kinetics, we prepared a line chart using the mean (n = 6) bacterial colony counts (log_10_ CFU/mL) and time (h) for each treatment.

Stability was assessed against *S. typhimurium* ATCC 14028 and *S. aureus* ATCC 25923 by agar well diffusion assay. The 70% (*v*/*v*) ethanolic extract was pre-exposed for 30 min to varying temperatures (37 °C, 70 °C and 100 °C) and pH (2, 4, 6 and 8), followed by the evaluation of inhibition zones. Additionally, the refrigerated (4 ± 1 °C) storage stability of the extract was evaluated after 0, 15 and 30 days of aging [17].

#### 2.2.6. In Vitro Toxicity Assays (Hemolysis Assay, Effect on Probiotics and Vero Cell Proliferation and Viability Assay)

The hemolysis assay was performed using sheep red blood cells (RBCs), as described by Ebbensgaard et al. [27]. Briefly, sheep blood (2 mL) was collected aseptically, washed several times with PBS, centrifuged at 1000× *g* for 15 min, and resuspended in 10% (*v*/*v*) PBS containing 10 mM dithiothreitol (DTT). Aliquots (100 µL) of formulation at concentrations equivalent to 1X, 2X, 3X, 5X, and 10X MIC against *S. aureus* ATCC 25923 were mixed with 100 µL of the RBC suspension. Sterile PBS and 0.2% (*v*/*v*) Triton X-100 served as negative and positive controls, respectively. Hemoglobin release was quantified spectrophotometrically at 540 nm using the following formula:

$$Hemolysis\;(\%)=\frac{Absorbance\;of\;Sample\;-\;Absorbance\;of\;PBS}{Absorbance\;of\;Triton\;X\;-\;Absorbance\;of\;PBS}\times 100$$

The effect of the formulation on the growth of probiotic lactobacilli (*Lactiplantibacillus plantarum* MTCC 2621 and *L. casei* MTCC 1423), which are natural commensals in the gut, was analyzed as before [26]. Briefly, the lactobacilli bacteria (100 µL each, 10^7^ CFU/mL) were inoculated into 100 µL of deMann–Rogosa–Sharpe (MRS) broth containing the formulation (1X MIC and 2X MIC for SA) in a 96-well microtiter plate. The untreated bacterial culture and MRS broth were included as positive and negative controls, respectively. After incubation at 37 °C for 48 h, the cultures were diluted in MRS broth, and 10 µL from each dilution was plated on MRS agar plates to enumerate colony-forming units to assess the formulation effect on the probiotics.

The effect of formulation, if any, on Vero cells (monkey kidney epithelial cell line; ATCC CCL-812) was measured by a 3-(4.5-dimethylthiazolyl-2)-2, 5-diphenyltetrazolium bromide (MTT) assay. Vero cells (5000 cells/well) were seeded in a 96-well microtiter plate, cultured in Dulbecco’s Modified Eagle Medium (DMEM), supplemented with 10% heat-inactivated fetal bovine serum (FBS), 1% antibiotic (100 IU/mL penicillin), and antifungal (100 IU/mL streptomycin) solution. The plates were incubated at 37 °C for 24 h in a controlled atmosphere of 5% CO_2_ and 95% air. Thereafter, the cells were treated with different concentrations of the formulation (2.5, 5, 7.5, and 10%, *w*/*v*) for 48 h. The positive control was H_2_O_2_ (500 µM), while culture medium alone served as the negative control. Subsequently, 15 µL of MTT (stock concentration = 4 mg/mL) was added to each well to give a final concentration of 500 µg/mL, and the mixture was incubated for 24 h. After 24 h, the unused DMEM and remaining MTT reagent were removed by aspiration. The formazan crystals formed were dissolved with 80 µL of dimethyl sulfoxide per well. The optical density (OD) at 570 nm was measured using a microplate reader (SpectraMax^®^ M5, Molecular Devices, San Jose, CA, USA). The percent cell viability, as compared to the untreated control, was calculated by the following formula:

$$\%\;Cell\;Viability=\frac{OD\;of\;Treatment}{OD\;of\;Negative\;Control}\times 100$$

### 2.3. Preparation and Quality Evaluation of Chicken Sausages

Broiler chickens were slaughtered, dressed, and deboned according to standard procedures at our institutional meat processing facility. Meat was frozen at −18 ± 2 °C, later thawed at 4 ± 1 °C, and then minced through 8 and 4 mm plates. Batches were mixed (2–3 min) in a bowl chopper to form a fine emulsion (batter at 15 °C), stuffed (500 g per batch) into 24 mm artificial casings to yield 50 g sausages (~10 cm), and cooked in a water bath at 80 °C for 30 min (internal temperature 75 ± 1 °C). The sausages were cooled to room temperature, aerobically packaged in low-density polyethylene (LDPE) pouches, and stored at 4 ± 1 °C for 20 days. In total, five test chicken sausage groups, i.e., negative control (C1) containing no phytochemical or nitrite, positive control (C2) containing no phytochemical but 150 ppm nitrite, T1B (0.25% formulation + 75 ppm nitrite), T2B (0.5% formulation + 50 ppm nitrite), and T3B (1% formulation + no nitrite), were prepared. The five test groups of emulsion-based cooked chicken sausages were analyzed for expressible water content, proximate composition, and calorific value on the day of processing. Quality changes in the test chicken sausages were evaluated during refrigerated storage (4 ± 1 °C) under aerobic packaging for 20 days at 5-day intervals (day 0, 5, 10, 15, and 20).

#### 2.3.1. Expressible Water, Proximate Composition, and Calorific Value

The expressible water (%) in each sausage group was determined by the method of Jauregui et al. [28]. About 5 g of sample was placed between two layers of Whatman no. 1 filter paper (GE Healthcare, Waukesha, WI, USA). The sample was centrifuged at 1500× *g* for 15 min (Remi, Mumbai, Maharashtra, India). The final weight was recorded, and the expressible water (%) of the sample was calculated as follows:

$$Expressible\;water\;(\%)=\frac{Initial\;weight\;\left(g\right)-Final\;weight\;(g)}{Initial\;weight\;\left(g\right)}\times 100$$

The proximate composition, such as moisture, crude protein (CP), crude fat (CF), and total ash (TA) of the sausage samples, was analyzed as per AOAC [18]. The carbohydrate contents (%) were estimated by difference.

$$Carbohydrate\;\left(\%\right)=100-(Moisture\;\%+CP\;\%+CF\;\%+TA\;\%)$$

The calorific values (kcal/100 g) of sausage samples were calculated using the Atwater values for CF (9.00 kcal/g), CP (4.02 kcal/g), and carbohydrate (4.00 kcal/g).

Calorific Value (kcal/100 g)=(CF%×9)+(CP%×4.02)+(Carbohydrate %×4)

#### 2.3.2. Thiobarbituric Acid-Reacting Substances (TBARS) Values, Residual Nitrite (RN) Content, and Shear Force Value

The TBARS values of the sausage samples were determined according to the method by Witte et al. [29] with slight modification and expressed as mg malonaldehyde per kg (mg MDA/kg) of the sample. The RN content of sausage samples was determined by the slightly modified method of AOAC [18], as already described in Section 2.2.1. The shear force values of sausage samples were measured instrumentally by a shear force apparatus (SHD-27, Superb Technologies, Haryana, India) at all specified storage intervals as per the slightly modified procedure of Berry and Stiffler [30] using the following formula: *Shear force value* (kgf/cm^2^) = *Kilogram-force required to shear the sausage* (kgf)/*Cross-sectional area of the sausage* (cm^2^).

#### 2.3.3. Total Pigment, Cured Pigment and Curing Efficiency

Nitroso/cured pigment (nitrosohemochrome) and total pigment in sausage samples were estimated following the method of Hornsey [31]. Cured pigment concentration (mg/kg) was calculated using the absorbance at 540 nm (A_540_) by the formula

$$Cured\;(Nitroso)\;pigment\;(mg/kg)=A_{540}\times 290.$$

Total pigment concentration (mg/kg) was calculated using the absorbance at 640 nm (A_640_) by the formula

$$Total\;pigment\;(mg/kg)=A_{640}\times 680.$$

The % conversion or curing efficiency (%) was calculated by the formula

*Curing efficiency* (%) = [*Cured pigment* (mg/kg)/*Total pigment* (mg/kg)] × 100

#### 2.3.4. Total Viable Count (TVC), Psychrophilic Count, Coliform Count, and *S. aureus* Count

The total viable count (TVC), psychrophilic count, coliform count, and *S. aureus* count of the sausage samples were estimated at specified storage intervals by the pour plate method, as described by APHA [32]. Briefly, 10 g of sausage sample was transferred aseptically into a stomacher bag (Seward Medical, Newport Gwent, Wales, UK) containing 90 mL of 0.1% sterile buffered peptone water (BPW) and homogenized for 2 min. The samples were serially diluted in BPW, pour-plated with 1 mL of diluent, and incubated. Plate-count agar (PCA) was used for TVC and psychrophilic counts; violet red bile agar (VRBA) and Baird-Parker agar (BPA) were used for coliform and *S. aureus* counts, respectively, and incubated at 37 ± 1 °C under aerobic conditions for 48 h and 24 h. For psychrophilic counts, plates were inverted and incubated at 4 ± 1 °C for 7 days. Plates showing characteristic bacterial colonies were counted, and the average number of colonies was multiplied by the reciprocal of the dilution factor, then expressed as log_10_ CFU/g of sample.

#### 2.3.5. Sensory Attributes

Sensory evaluation of the sausage samples was performed at specified storage intervals using 10 semi-trained panelists and an 8-point hedonic scale [25].

### 2.4. Statistical Analysis

Data for each parameter were recorded in duplicate (analytical replicates), and the current study was replicated three times (n = 6 for each parameter, except for sensory evaluation, n = 30). Duncan’s multiple-range test and one-way and two-way analyses of variance (ANOVA) were used to analyze the data using the Statistical Package for the Social Sciences (SPSS, IBM, NY, USA, Version 20) software. The level of significance was set at 95% (*p* ≤ 0.05) for all parameters.

The ANOVA model considered treatment and storage period as the principal fixed factors, with their interaction evaluated where applicable. Before analysis, data were assessed for the assumptions of ANOVA, including the approximate normality of residuals, homogeneity of variance, and independence of experimental observations. One-way and two-way ANOVA were applied as appropriate, followed by Duncan’s multiple-range test for comparison of means. Statistical significance was considered at *p* ≤ 0.05.

## 3. Results

### 3.1. Preparation of Formulation Blend

Preliminary screening results for stability, color development, antioxidant activity, and antimicrobial activity (expressed as zone of inhibition (ZOI, mm)) were used to evaluate the properties of phytochemical blends. No significant differences (*p* > 0.5) were observed among the formulations consisting of RFP, MFP, and CmEO at different ratios (4:4:2; 5.5:4:0.5; 5:4:1) with respect to stability, color development, or antioxidant capacity. However, the ZOI values differed significantly (*p* ≤ 0.05; Supplementary Table S1), and all three combinations exhibited enhanced antimicrobial effects compared with their individual components. Of the three initial formulations, the blends with ratios 5.5:4:0.5 and 5:4:1 showed the highest antimicrobial activity, and the 5.5:4:0.5 blend (designated “formulation”) was selected for further studies. Although the antimicrobial activities of the 4:4:2 and 5.5:4:0.5 formulations were statistically similar, the latter was chosen because it had a lower proportion of cinnamon EO, which was desirable from a sensory standpoint. It is well-established that no single nitrite substitute has all the properties needed to completely replace it in processed meat products [11]. Therefore, researchers have also used blends of EOs, combinations of plant extracts with EOs, and combinations of spice extracts as nitrite replacements in emulsion-based chicken sausages [33] and fermented sausages [14,34].

### 3.2. Characterization of Phytochemical Formulation

#### 3.2.1. Total Extractable Components (TEC), pH, Color and RN Content of Formulation

The TEC (%) of our chosen formulation was 26.79% (Table 1), indicating its improved solubility and extractability. The individual plant powders present in the formulation could have affected TEC or yield. In another study, researchers also calculated the yield (%) of tomato pomace (4.77%) by supercritical fluid extraction and peppermint EO (0.70%) by hydro-distillation, and used them as natural nitrite replacements in cooked pork sausage [6]. Variations in yields could be attributed to many factors, such as the extraction method used, the type of solvent, the plant derivative, the plant’s growth stage or maturity, environmental conditions, and seasonal growing conditions [35,36].

The mean pH of the formulation was 3.28 (Table 1), indicating its acidic nature and supporting its use in low-acid foods, such as meat products. As reported in earlier studies, extracts of individual plant powders, such as RFP (pH, 2–3) and MFP (pH 5.44), are acidic [36,37]. The low pH of the phytochemical combination may be due to the acidic nature of the individual constituents (RFP and MFP) used in this study.

The CIELAB instrumental color values of the formulation were L* (21.87), a* (19.88), b* (13.43)*,* h_ab_ (6.20) and C*_ab_ (25.01) (Table 1). In general, the L* value ranges from 0 (black) to 100 (white), whereas a* values, if positive, indicate redness and negative values indicate greenness. Likewise, negative b* values represent bluish color, whereas positive b* values correspond to yellowness. The b*, h_ab_ and C*_ab_ values are positively correlated [38], where h_ab_ stands for hue angle/tonality and C*_ab_ indicates color vividness and a bright reddish nature. In parallel with our study, Ozaki et al. [14] also measured the color parameters of radish powder (L* = 60.03, a* = 17.24, b* = 0.27) and beetroot powder (L* = 30.17, a* = 16.90, b* = 2.94), which were used as natural nitrite replacements in dry-fermented pork sausages.

No RN content was detected in our formulation, as evidenced by the absence of pinkish-violet color development following the addition of N-(1-naphthyl) ethylenediamine dihydrochloride (NEDA) reagent to the reaction mixtures, in contrast to the nitrite-positive control (Table 1). These findings corroborate those of Bahadoran et al. [39], who reported undetectable levels of nitrite and nitrate in common fruits and vegetables, including tomato, carrot, cucumber, and potato. The lack of residual nitrite in the phytochemical components of our formulation may be attributed to organic cultivation practices and the absence of nitrogenous fertilizers during plant growth [8].

#### 3.2.2. Antioxidant Activity Assays (TPC and DPPH Assay)

In this study, we observed significant (*p* ≤ 0.05) differences between the mean TPC of our formulation (147.44 mg GAE/g) and synthetic standard antioxidants such as BHT (2.82 mg GAE/g) and BHA (3.25 mg GAE/g) (Figure 1A). The TPC of the formulation can be attributed to the presence of polyphenols, phenolic acids, flavonoids, saponins, tannins, and alkaloids, among other components [40]. Similar to our study, Ozaki et al. [14,34] estimated the TPC of radish (301.11 mg GAE/100 g), beetroot (292.96 mg GAE/100 g) powders, and oregano EO (62 mg GAE/100 g), and used them as natural nitrite replacers in fermented sausages. In another study, Aquilani et al. [41] determined the TPC of grape seed extract (822.709 mg/g), chestnut extract (161.091 mg/g), and olive pomace hydroxytyrosol (32.62 g/L), and used them in combination as a natural sodium nitrite substitute in Cinta Senese dry-fermented pork sausage. The differences in the total phenolic content of natural plant ingredients can be attributed to several factors, including plant part type, climate, geographic conditions, extraction method, and solvent type [42].

The DPPH assay measures the electron-donating activity of a substance by scavenging or reducing DPPH free radicals and is a measure of its antioxidant activity [43]. The data revealed a significant difference (*p* ≤ 0.05) in the mean IC_50_ values of ethanolic extracts of our formulation (63.81 µg/mL) as compared to BHT (13.12 µg/mL), BHA (6.99 µg/mL), and sodium nitrite (31.14 µg/mL) (Figure 1B). The DPPH radical-scavenging activity of the formulation could be attributed to its individual components, including plant phenolic hydroxyl groups (catechol, hydroquinone, etc.) that donate an electron to neutralize DPPH free radicals [44].

Šojić et al. [6] also estimated the DPPH scavenging activities of tomato pomace (30.82 µM TE/g) and peppermint EO (23.16 µM TE/g), which they used as natural nitrite replacements in cooked pork sausage. Similarly, Ozaki et al. [14,34] estimated the DPPH radical-scavenging activities of the powders of radish (1537.65 mg TE/g), beetroot (7140 mg TE/g), and oregano EO (6210 mg TE/100 g), and used them as natural nitrite replacements in fermented pork and beef sausages. The differences in DPPH assay results might be due to various factors, as mentioned earlier for TPC.

#### 3.2.3. Preliminary Identification of Phytochemical Compounds Through GC–MS

The GC–MS/MS chromatogram revealed the presence of 30 major phytochemical compounds in the formulation, which have been tentatively identified (Supplementary Figure S1 and Supplementary Table S2). The compounds were mostly secondary plant metabolites, including polyphenols, ethers, esters, carboxylic acids, alcohols, aldehydes, ketones, and aromatic derivatives. Their occurrence likely reflects both the intrinsic phytochemical profiles of cinnamon essential oil and roselle and moringa flower powders, and potential interactions among these components during formulation. Several compounds were preliminarily detected at relatively high proportions, suggesting their potential contribution to the functional properties of our formulation. For instance, 1-Octanol, 3,7-Dimethyl- (citronellol) was the most abundant constituent, followed by Cyclohexane, 1,2-Dimethyl-, cis- (cis-dimethyl cyclohexane), 6-Methyl-Cyclohex-2-En-1-Ol (carveol isomer), and Phosphonic acid, 7-octenyl-, dibutyl ester. Other notable compounds included 1,3,5-Tris(cyclohexyl)pent-1-ene, Cyclohexane, 1,1′-(2-propyl-1,3-propanediyl) bis-, and 2,5-Furandione, 3-dodecyl-. These phytochemical compounds may have contributed to the acidic pH and the antioxidant and antimicrobial activities observed in the present study.

Furthermore, the GC–MS results here represent an exploratory analysis of detectable volatile and semi-volatile compounds, rather than a complete phytochemical profile of the formulation, which can be conducted using complementary approaches such as LC–MS/MS, NMR spectroscopy, confirmation using authentic standards, etc. Similar to our study, Šojić et al. [45] also chemically characterized sage EO by GC–MS, identified several terpenoids and phenolic compounds, and used them as potential natural nitrite replacements in dry-fermented pork sausage. Šojić et al. [6] and Ozaki et al. [34] also identified phenolic compounds and terpenoids in peppermint EO (menthon, menthol, isomenthon, eucalyptol, etc.) and oregano EO (carvacrol, p-cymene, γ-terpinene, and thymol), which were used as natural nitrite replacements in cooked pork sausages and fermented cooked sausages, respectively.

#### 3.2.4. Agar Well Diffusion Assay, MIC, MBC and Tolerance Level

The formulation exhibited strong antimicrobial activities against several foodborne pathogens, including *S. aureus* ATCC 25923 (18.5–30.5 mm), *L. monocytogenes* ATCC 19111 (18.41–30.58 mm), *L. monocytogenes* ATCC 13932 (24.67–30.58 mm), *S. typhimurium* ATCC 14028 (18.33–2.08 mm), *E. coli* ATCC 25922 (14.58–20.67 mm), *C. sporogenes* MTCC 2684 (30.92–32.91 mm) and *C. perfringens* MTCC 450 (22.83–24.33 mm) in agar well diffusion assay (Figure 2). The formulation showed significant differences (*p* ≤ 0.05) in ZOI at lower and higher concentrations against all target bacteria. These antimicrobial activities can be attributed to the inherent bioactive compounds and enhanced antibacterial effects of the individual components.

In this study, the penultimate concentration (10%) showed significantly greater antimicrobial activity than the lower (5%) and higher (20%) concentrations, suggesting it was optimal. One possible reason is that the lower concentration (5%) may be insufficient, whereas a higher concentration (20%) could cause compound aggregation, reducing bioavailability, increasing toxicity, and resulting in lower antimicrobial activity than the penultimate concentration [46]. Das et al. [25] observed a similar trend and reported that bamboo essential oil (BEO) exhibited broad-spectrum antibacterial activity against *S. aureus* (2.48 cm), *S. typhimurium* (2.8 cm), and *E. coli* (1.78 cm). Researchers have also reported broad-spectrum antimicrobial activity of individual plant components, such as roselle flower [47], moringa flower [48], and CmEO [49], or the synergistic effects of their combination (plant extracts and EOs) [50].

The MIC was determined as the lowest concentration of the formulation that prevented the resazurin dye from changing from blue to pink [51]. After overnight incubation, MBC was recorded as the lowest extract concentration yielding no visible colonies on MHA plates [25]. The data summarized in Table 2 indicate that there were significant (*p* ≤ 0.05) differences in values for MIC, MBC, and tolerance level between Gram-positive (*S. aureus* ATCC 25923, *L. monocytogenes* ATCC 19111) and Gram-negative (*S. typhimurium* ATCC 14,028 and *E. coli* ATCC 25922) bacteria used in this study. The MIC and MBC of the formulation for both Gram-positive bacteria were the same (0.781 mg/mL), and the tolerance level was 1. However, the results showed different values of MIC, MBC, and tolerance level for *S. typhimurium* ATCC 14028 (MIC = MBC = 0.195 mg/mL and 1) and *E. coli* ATCC 25922 (0.39 mg/mL, 0.781 mg/mL, and 2). Furthermore, the positive control, ciprofloxacin, showed the same MIC, MBC, and tolerance levels for each bacterium. However, there were significant differences (*p* ≤ 0.05) among the values for Gram-positive (0.125 mg/mL, 0.25 mg/mL, and 2) and Gram-negative bacteria (0.25 mg/mL, 0.5 mg/mL, and 2). The lower MIC, MBC, and tolerance levels suggest the formulation’s potent antimicrobial activity.

In a study, Sharma et al. [52] also determined the MBC of nine EOs against seven foodborne bacteria and evaluated their use as preservatives in vacuum-packaged chicken sausages under frozen storage conditions. The MIC, MBC, and tolerance levels of BEO [25] and *Alkanna tinctoria* roots [17] were also determined against several common foodborne bacteria before using them as natural preservatives in chicken meatballs. Generally, a low (≤4), high (≥4), and very high (≥16) MBC/MIC ratio or tolerance level indicates that the plant extract or antibiotic is bactericidal, bacteriostatic, and tolerant, respectively [25]. The low tolerance levels or MBC/MIC ratios (≤4) of our formulation and ciprofloxacin suggest that both have bactericidal activity, i.e., can kill the bacteria at concentrations closer to or equal to MIC.

#### 3.2.5. Bacterial DNA Degradation

The DNA-degrading bioactivity of the phytochemical formulation was assessed via agarose gel electrophoresis (Figure 3). The treatment of plasmid DNA (pUC19) and *S. aureus* ATCC 25923 genomic DNA (gDNA) with the ethanolic extract of the formulation induced degradation, converting double-stranded circular plasmid DNA (lane 3) and genomic DNA (lane 7) into fragmented forms. In contrast, DNase treatment completely degraded both plasmid (lane 4) and gDNA (lane 8), serving as a positive control (Figure 3).

These results indicated that the formulation contained bioactive compounds that degraded extrachromosomal plasmid DNA (pUC19) and *S. aureus* (ATCC 25923) genomic DNA, thereby contributing to their antimicrobial activities. Moreover, plasmid DNA degradation is an important finding from an antimicrobial resistance (AMR) perspective, as plasmids contribute to multidrug resistance (MDR) by harboring and disseminating multiple resistance genes [53]. Das et al. [26] reported similar results in their study on the antioxidant and antimicrobial activities of *Sesamum indicum* honey.

#### 3.2.6. Dose- and Time-Dependent In Vitro Growth Kinetics and Stability Assays

The time-kill assay results show dose- and time-dependent in vitro growth kinetics of *S. aureus* (ATCC 25923) (Figure 4). From the findings, it is apparent that the untreated control exhibited an increasing growth pattern (7.56–9.51 log_10_ CFU/mL) throughout the incubation period (i.e., up to 24 h). In contrast, the antimicrobial effect of ciprofloxacin at the 1X MIC (treatment control) was highly significant (*p* ≤ 0.01) at 3 h post-incubation (hpi) (4.53 log_10_ CFU/mL), with more than 3-log reductions in bacterial counts relative to the control. The bacterial count exhibited almost static growth (3.81–4.02 log_10_ CFU/mL) with 1X MIC of ciprofloxacin until 24 hpi, whereas the untreated control exhibited increased growth, i.e., 9.5 log_10_ CFU/mL at the same time point. Similarly, the antimicrobial effect of ciprofloxacin (treatment control) at 1X MBC was highly significant (*p* ≤ 0.01) at 3 hpi (3.35 log_10_ CFU/mL), with a much lower bacterial count than in all treatments. At 6 hpi, the treatment with 1X MBC of ciprofloxacin completely inhibited *S. aureus* ATCC 25923.

Our formulation showed significant (*p* ≤ 0.05) antimicrobial effects at 1X MIC and 1X MBC compared to the untreated control. However, at 1X MIC and 1X MBC, the antimicrobial effects were highly significant (*p* ≤ 0.05) from 3 hpi onward. Treatment with 1X MIC of the formulation reduced bacterial counts from 7.48 log_10_ CFU/mL to 4.48 log_10_ CFU/mL at 0–24 hpi. Moreover, we observed complete elimination of *S. aureus* ATCC 25923 at 9 hpi, with the formulation achieving 1X MBC. These kinetic studies demonstrate that our formulation is highly effective at the MBC concentration; however, a significant (*p* ≤ 0.05) reduction in log_10_ CFU/mL was also observed at its MIC concentrations.

In a study, Prasastha Ram et al. [54] also reported that 1X MBC-level of green-synthesized nanosilver entrapped with cinnamaldehyde eliminated MDR *E. coli* after 2 hpi. In a similar study, Olajuyigbe and Afolayan [55] reported that the ethanolic extracts of *Erythrina caffra* bark killed pathogenic bacteria, including *Micrococcus luteus*, *Proteus vulgaris*, and *S. aureus*, after 8 h of incubation at 2X MIC.

Our formulation showed excellent antimicrobial activity against *S. typhimurium* ATCC 14028 and *S. aureus* ATCC 25923 at moderate to high temperatures (37 to 100 °C), and under acidic conditions (pH 2 to 6), and remained stable up to 30 days under refrigerated storage conditions (Table 3). As temperature increased (37 °C, 70 °C, and 100 °C), ZOI decreased significantly (*p* ≤ 0.05) in both bacteria, but the formulation remained effective at all temperatures. Therefore, its use as a food preservative would be highly advantageous, as temperature plays a paramount role in food processing and preservation. The formulation showed high pH stability (adjusted using 1N HCl and 1N NaOH, Ref #15) and strong antimicrobial activity at acidic pH (2, 4, and 6), but lost activity at basic pH (8). Possible reasons for the loss of antibacterial activity at alkaline pH (8.0) include the degradation of natural bioactive compounds and changes in ionization states, which can decrease the solubility of active compounds and reduce interaction with target bacterial cells [56]. In addition, higher pH conditions could accelerate the degradation of phenolic and other bioactive compounds, thereby reducing functional activity. The highest activity was recorded at pH 4, and no significant (*p* > 0.05) differences in ZOI were observed at pH 4 and pH 6 for both selected bacteria, indicating that the formulation retained substantial activity across a mildly acidic range. This suggests its suitability for use in acidic animal-origin foods such as meat, fish, and milk-based products, where maintaining a lower pH can support antimicrobial performance and improve product safety. Significant (*p* ≤ 0.05) differences in the ZOI of the formulation were observed against *S. typhimurium* ATCC 14028, and non-significant (*p* > 0.05) differences in ZOI for *S. aureus* ATCC 25923, between 0-day and 15-day storage at 4 ± 1 °C. Although there were significant (*p* ≤ 0.05) differences in the ZOI against the test bacteria on day 0 and day 30 of refrigerated storage (4 ± 1 °C), appreciable antimicrobial activity was still observed on day 30. These results suggest that our formulation was stable under refrigerated storage and would be an attractive biopreservative and colorant in chilled foods.

In our previous study, it was also reported that the antimicrobial activity of ratanjot root extract was stable at varying temperatures (37–100 °C), pH conditions (2–6), and storage at refrigerated (4 ± 1 °C) conditions up to 30 days [17]. Similar stability assays to assess antimicrobial activity under varying conditions were conducted by Das et al. [57] for a crude extract from Indian curd against *B. cereus* and *S. typhimurium*, and by Prasastha Ram [54] for green-synthesized nanosilver with entrapped cinnamaldehyde against MDR enteroaggregative *E. coli*.

#### 3.2.7. Hemolysis Assay

An in vitro hemolysis assay is a simple, cost-effective alternative for assessing the in vitro cytotoxicity of phytochemicals on mammalian cells before recommending their use in human food and/or medicine [58]. If the hemolysis induced by a plant extract exceeds 30%, it is considered hazardous to red blood cells (RBCs) [59]. The results (Figure 5) show significant differences (*p* ≤ 0.05) in the degree of hemolysis caused by our formulation compared to the positive control (Triton X-100), which showed complete (100%) hemolysis with sheep RBCs. However, we observed no (0%) hemolysis at lower concentrations (1X, 2X, and 3X MIC of SA), and negligible hemolysis (<0.015% and <0.37%) at higher concentrations (4X and 5X MIC of SA), indicating it is non-cytotoxic. The anti-hemolytic properties of plant extracts might be due to their phenolic contents exerting antioxidant activity on RBC membrane rupture [60]. Similar anti-hemolytic activities of phytochemicals or extracts from several plants, such as *Portulacaria afra* [59] and mangrove plants [60], have also been reported.

#### 3.2.8. Effect on Probiotics

The probiotic safety evaluation showed no significant inhibitory effects on beneficial gut lactobacilli (Figure 6A). *L. casei* MTCC 1423 and *L. plantarum* MTCC 2621 maintained high mean viable counts when exposed to both 1X MIC and 2X MIC treatments, with values (9.27 ± 0.33 and 9.14 ± 0.21 log CFU/mL for *L. casei*; 9.40 ± 0.31 and 9.23 ± 0.19 log CFU/mL for *L. plantarum*) comparable to the positive control (9.31 ± 0.24 and 9.44 ± 0.21 log CFU/mL), respectively. Statistical analysis indicated no significant (*p* > 0.05) reduction in growth at 1X MIC, and only a marginal decrease at 2X MIC, confirming that the formulation does not adversely affect probiotic viability.

The findings in this study clearly indicate the selective inhibition of our formulation against the potentially harmful foodborne pathogenic bacteria, while sparing probiotic lactobacilli, a critical property for functional food applications, as it supports host gut health and microbial balance [61]. In fact, the phytochemical constituents of our formulation, including antioxidant dietary fibers, flavonoids, and phenolic compounds, may act as prebiotics and play a crucial role in promoting the growth and activity of probiotic gut lactobacilli [62], positively impacting host health. Such dual functionality, antimicrobial selectivity coupled with probiotic compatibility, makes it an ideal “clean-label” or natural alternative to chemical additives.

Similar non-inhibitory or growth-promoting effects have been reported for plant extracts such as *Achillea millefolium* L. (yarrow) herb extract [62], *Cannabis sativa* L. or hemp seed extracts [61], and *Sesbania grandiflora* or agasti flower extract [63]. These studies collectively confirmed that phytochemical-rich formulations can simultaneously suppress pathogenic bacteria while sustaining or even promoting probiotic populations such as *L. plantarum*, *L. acidophilus*, *Lacticaseibacillus rhamnosus*, *Bifidobacterium bifidum*, and probiotic yeasts.

#### 3.2.9. Vero Cell Proliferation and Viability Assay

We performed an MTT assay to assess cell proliferation and viability to evaluate the toxicity of a new phytochemical before incorporating it into food systems. The mean % Vero cell viability for our formulation (2.5–10%, *w*/*v*) ranged from 86.15 to 95.80% (Figure 6B). Further, only small amounts of cell death were observed in a dose-dependent manner after 24 h of incubation. In contrast, the positive control (500 µM H_2_O_2_) and negative control yielded 3.48% and 100% cell viability, respectively, suggesting it is non-toxic to mammalian cells. Given this favorable non-cytotoxicity profile at substantially higher test concentrations, the much lower level used in our chicken sausages (≤1% phytochemical formulation) is expected to pose no cytotoxic risk to consumers. In an MTT assay, Das et al. [42] also found that clove EO nanoemulsion was non-cytotoxic in a meat model system at 2.5–100 µL/mL, even after 48 h of incubation. Several studies have also evaluated the cytotoxicity aspects of different plants, such as *Cassia surattensis* [64] and *Euphorbia helioscopia* [65], using Vero cells in an MTT assay.

### 3.3. Quality Evaluation of Chicken Sausages

#### 3.3.1. Expressible Water, Proximate Composition and Calorific Value

The expressible water is a measure of the water-holding capacity (WHC) of emulsion-based meat products. The lower expressible water indicates higher product WHC [17]. The mean expressible water (%) of the control and our formulation-treated samples ranged from 22 to 25% (Table 4). C1 significantly (*p* ≤ 0.05) showed higher expressible water (%) compared to C2 and other treated products, whereas T3B showed lower values than the other test groups, indicating its higher WHC. This may be due to greater water-binding by the phytochemical formulation in the treated sausages, compared to the control. These results agree with those of Das et al. [25,35], who reported similar values for expressible water content in goat meat nuggets incorporated with moringa pods (19.25–21.85%) and in chicken meat balls with BEO (25.72–26.96%).

The % moisture, CP, CF, TA, and carbohydrate contents of the control and treated chicken sausages using our formulation were in the ranges of 66.02–67.24%, 15.30–16.07%, 12.14–13.04%, 2.09–2.32%, and 2.76–2.98%, respectively (Table 4). Conducting proximate composition analysis on emulsified pork sausages containing red beetroot powder as a natural nitrite substitute, Jin et al. [66] reported moisture (67.01–68.8%), CF (11.63–13.84%), crude ash (0.48–1.68%), and CP (17.13–17.58%). In another experiment, Šojić et al. [45] reported a slightly different proximate composition of moisture (31.5–35%), protein (22.9–28.9%), fat (31.1–37.4%), and ash (5–5.8%) for dry-fermented sausages incorporated with sage EO as a nitrite replacement.

The mean calorific values of the control and treated chicken sausages using our formulation ranged from 182.72 to 193.09 kcal/100 g (Table 4). The treated sausages had lower calories than the control sausages because we substituted a portion of the meat with phytochemical combination powders, making them healthier from a consumer health perspective. Similar to our study, Moirangthem et al. [67] also reported that the caloric values of duck meat sausages containing soy protein isolate and inulin ranged from 165.94 to 180.00 kcal/100 g. Differences in expressible water content, proximate composition, and calorific value among different meat products observed by various researchers could be attributed to differences in product formulations, ingredient amounts and types used, emulsion quality, cooking methods, etc. [68].

#### 3.3.2. Thiobarbituric Acid-Reactive Substances (TBARS) Values

The mean TBARS values (mg MDA/kg) for C1 and C2 ranged from 0.287 to 2.031 and 0.286 to 1.051, respectively, whereas the mean values for phytochemical formulation-treated chicken sausages were T1B (0.278–0.941), T2B (0.281–0.773), and T3B (0.280–0.761). As storage days increased, mean TBARS values in chicken sausages increased significantly (*p* ≤ 0.05) (Table 5). This might be due to lipid oxidation and the production of volatile metabolites in the presence of oxygen under aerobic packaging conditions [36]. However, the rate of increase in TBARS values in the sausages (T1B, T2B and T3B) was slower, which might be due to the high phenolic content and antioxidant activity of the phytochemical formulation [36]. Furthermore, the mean TBARS values of the treated sausages remained below the acceptable level (1–2 mg MDA/kg) throughout the storage period [25]. These findings are consistent with the results of Huang et al. [69], who reported an increasing trend of TBARS values in control (0.16–1.47, 0.71–2.05) and treated cured meat (0.35–1.46) with nitrite substitutes like *Monascus* color, beet red, nisin, and starter cultures of *L. fermentum* RC4 and *L. plantarum* B6 during 20 days of refrigerated storage. Jin et al. [70] also reported an increase in TBARS value (0.62–1.52 mg MDA/kg) of pork sausages, prepared with a combination of 0.04% paprika powder and 0.03% blueberry powder as a natural nitrite replacement during 4 weeks of refrigerated storage.

#### 3.3.3. Residual Nitrite (RN) Content

The RN contents (mg/kg) of the control and our formulation-treated chicken sausages (Table 5) show that the ranges of mean RN contents (mg/kg) of C2, T1B, and T2B are 80.35–46.95, 47.14–26.46, and 31.10–16.03, respectively. The nitrite contents of test sausage samples except C1 on day 0 were within the maximum permissible limit of 80 mg/kg for comminuted, processed meat products, as set by the Codex Alimentarius Commission [71]. The slightly higher value observed for C1 on day 0 is a Mean (n = 6) ± SE value; this was very close to the permissible limit and might have resulted due to sampling or analytical errors. As no pink color development was evident in the reaction mixture containing C1 and T3B samples by the analytical method followed, and no limit of detection (LOD) and limit of quantification (LOQ) were determined, it was considered that RN contents were below detection capability in those samples. This may be due to the lack of sodium nitrite in the formulation, the absence of nitrite contamination during product processing [14], or the absence of nitrite in the individual ingredients, such as the phytochemical combination and chicken meat due to improved feeding and management [8]. We obtained significantly (*p* ≤ 0.05) lower RN contents for treated sausages (T1B and T2B) compared to a positive control (C2), which could be due to the reaction of highly reactive nitrite with different bioactive compounds (polyphenols, flavonoids, terpenes, etc.) present in the phytochemical combinations [5]. Further, the lower RN values in sausages than in the added nitrite might be due to losses during processing and to the reaction of chemical nitrite with meat proteins, lipids, myoglobin, and other constituents [72], as well as oxidation to nitrate, utilization by microbes and enzymes, etc. [5].

These results are in complete agreement with Šojić et al. [5], who reported no RN in the control pork sausages, 45–20 mg/kg RN in cooked pork sausages prepared with different concentrations of CEO and 50 mg/kg added nitrite, and 90–25 mg/kg RN in pork sausages with 100 mg/kg added nitrite during 60 days of storage at 4 °C. These findings also bear similarity with those of Ozaki et al. [14], who reported no RN content in control or sausages treated with 0.5% beetroot powder, but significantly (*p* ≤ 0.05) lowered RN content with 1% beetroot (7.75–1.48 mg/kg) or radish (17.11–9.9 mg/kg) powder-treated sausages as compared to the positive control C1 (93.15–1.19 mg/kg), and also observed a decreasing trend in the RN content of sausages with the advancement of processing for 35 days.

#### 3.3.4. Shear Force Value

The mean shear force values (kgf/cm^2^) for C1, C2, T1B, T2B and T3B were 1.88–1.44, 1.85–1.58, 1.84–1.59, 1.81–1.64, and 1.80–1.64, respectively, from day 0 to day 20 of storage (Table 5). Although a decreasing trend was observed throughout the storage period, significant differences (*p* ≤ 0.05) were observed between the control and treated sausages. The C1 sample had significantly higher (*p* ≤ 0.05) values during the initial storage days, indicating greater hardness. However, during the latter part of storage, significantly (*p* ≤ 0.05) lower shear force values were obtained, which may be due to the development of softness and poor texture resulting from microbial and enzymatic decomposition. In a study, Xin et al. [2] reported decreasing trends in shear force (N) values of cured mutton samples during refrigerated storage from day 0 to day 15, when PP extract and LEO were used as natural nitrite replacements. In partial agreement with our results, increased shear force values due to the addition of dried carrot pomace in chicken sausages (5.01–5.39 N) during a 15-day storage study have also been reported [73].

#### 3.3.5. Total Pigment, Cured Pigment, and Curing Efficiency

The cured meat pigment, total pigment, and curing efficiency of the test chicken sausages are summarized in Table 5. Total pigment ranged from 65.84 to 95.26 mg/kg and from 50.25 to 84.03 mg/kg on day 0 and day 20 of storage, respectively. However, sausages treated with our formulation had significantly higher (*p* ≤ 0.05) total pigment levels than the control, likely due to the excellent colorimetric properties of the phytochemical combinations.

Cured pigment levels in the control and treated sausages using our formulation ranged from 16.41 to 7.63 mg/kg on day 0 and from 5.59 to 49.34 mg/kg on day 20 of storage. As expected, the lowest (*p* ≤ 0.05) cured pigment was observed in a negative control (C1) and the highest (*p* ≤ 0.05) for the positive control containing 150 ppm nitrite (C2). The probable reason for the small amounts of cured pigment observed in nitrite-free sausage samples (C1 and T3B) could be attributed to some amount of cured pigment formation by other salts, like sodium chloride (common salt) or sodium tripolyphosphate used in the formulation, and to some complex biochemical reactions during product processing and storage. Previous studies detected cured pigments in fermented sausages, even without the addition of nitrite [14,74]. The reason could be that the nitroso pigment (nitrosohemochrome), formed by the reaction of nitrous oxide (NO) and myoglobin in meat at pH 5–6.5, is the principal cured pigment and responsible for the characteristic reddish-pink color of cured meat products [75].

Curing efficiency measures the conversion of total pigments to cured or nitroso pigments, and a higher conversion rate implies greater redness [74]. The mean curing efficiency (%) of the test sausages ranged from 6.65% to 69.42% on day 0 and day 20 of storage, respectively. The chicken sausage group (C2) had significantly higher (*p* ≤ 0.05) curing efficiency, primarily due to the addition of sodium nitrite, which is known to drive curing efficiency. Generally, well-cured meat using chemical nitrite has a curing efficiency of ≥ 80% [76]. However, sausage containing 150 ppm nitrite (C2) showed a slightly lower value (69.15–69.73%) than well-cured meat throughout storage. In contrast, T3B and C1 recorded significantly lower (*p* ≤ 0.05) curing efficiencies, which may be due to the absence of chemical nitrite. In these nitrite-free formulations, the phytochemical formulation’s intrinsic color likely contributed to the observed red hue, thereby influencing the apparent cured color development in T3B and C1 sausages [14]. Overall, cured and total pigments decreased, and curing efficiency declined with increasing storage days, which could be attributed to lipid–protein oxidation, oxygen presence, and microbial activity leading to color fading [14].

These results partially agree with Ozaki et al. [14], who reported similar cured pigment (3.84–53.94 kg/mg and 6.53–33.21 kg/mg), total pigment (66.30–264.52 mg/kg and 84.83–265.71 mg/kg) and curing efficiency (11.01–26.45% and 9.35–27.67%) in dry-fermented sausages using beetroot and radish powders as natural nitrite replacers with the advancement of processing and storage days. Serdaroğlu et al. [74] also reported nearly similar cured pigment (24.77–157.66 ppm), total pigment (109.35–197.17 ppm), and curing efficiency (22.65–83.52%) for control and treated fermented sausages, using arugula leaf extract and barberry extract as natural nitrite substitutes.

### 3.4. Microbiological Qualities of Sausages

#### 3.4.1. Total Viable Count (TVC)

TVC measures the live mesophilic bacterial load under aerobic conditions and is an important indicator of a product’s overall microbiological quality [17]. In this study, TVC differed significantly (*p* ≤ 0.05) between treated and control samples, and microbial growth increased with storage days (Table 6). The mean TVC (log CFU/g) of the control (C1 = 1.58–4.65, C2 = 1.41–4.13) and treated chicken sausages using our formulation (T1B = 1.38–3.81, T2B = 1.30–3.69, T3B = 1.28–3.52) from day 0 to day 20 of refrigerated storage are summarized in Table 6. On day 20, microbial counts in the control cooked chicken sausages exceeded the acceptable TVC limits (3–4 log_10_ CFU/g), as per the FSSAI [77] standards. However, treated cooked chicken sausages remained microbiologically safe and acceptable for up to 20 days, which might be attributed to the strong antimicrobial activity of the phytochemical formulation, including polyphenols, flavonoids, and tannins. Similarly, Ozaki et al. [14] reported reduced counts of mesophilic bacteria in dry-fermented sausages treated with 0.5% radish powder, as natural nitrite replacements. Several researchers have also reported lower TVC in various meat products containing natural nitrite replacers during storage and processing [5,34,78], compared with the control.

#### 3.4.2. Psychrophilic Bacterial Count

The mean psychrophilic counts (log CFU/g) of the control (C1 = 1.21–3.56, C2 = 1.29–2.41) and sausages treated with the phytochemical formulation (T1B = 1.17–1.94, T2B = 1.45, T3B = 1.24) are summarized in Table 6. During the initial days (0 and 5), psychrophilic counts were below the limit of detection in all groups, probably due to the efficacy of heat treatment during processing. Thereafter, psychrophile growth increased from day 10 onwards in C1 and from day 15 onwards in T1B, likely because these organisms prefer refrigeration temperatures during storage. However, lower psychrophilic counts in the treated chicken sausages than in the control may be due to the phytochemical formulation’s lower pH and antimicrobial activity, which make conditions unfavorable for psychrophile growth.

#### 3.4.3. Coliform Count

No coliforms were detected in any sausage samples throughout the storage period, suggesting the absence of fecal contamination during processing and storage [79]. Similar to our findings, no coliforms were reported in chicken fingers coated with clove, cinnamon, and thyme EO nanoemulsions [49], or in chicken nuggets with pomegranate seed powder, grape seed extract, and tomato powder [79] throughout the storage period. In contrast, higher coliform counts (<3 log CFU/g) were reported in control and treated dry-fermented sausages with radish and beetroot as natural nitrite replacements [14].

#### 3.4.4. *Staphylococcus aureus* Count

The *S. aureus* count in a food product indicates contamination from food handlers and inadequately cleaned food-contact surfaces [80]. The mean *S. aureus* counts (log CFU/g) of the control sausages (C1 = 1.30–2.55, C2 = 1.19–2.0) and our formulation-treated sausages (T1B = 1.04–1.78, T2B = 1.51, T3B = 1.34) are presented in Table 6. *S. aureus* was not detected during the initial days of storage. On day 15, control chicken sausages exceeded the maximum limit for *S. aureus* count (1–2 log_10_ CFU/g), as set by the Food Safety and Standards Authority of India (FSSAI) [77]. The treated sausages contained *S. aureus* within acceptable limits at the end of storage, attributable to the antimicrobial activity of our formulation. These results are consistent with Ibrahim et al. [80], who reported similar *S. aureus* counts for chicken nuggets (1.6 × 10^3^ CFU/g) and chicken shawarma (1.97 × 10^3^ CFU/g). In contrast, Huang et al. [69] detected no *S. aureus* in no-added-nitrite cured meat using natural nitrite substitutes.

### 3.5. Sensory Attributes of Sausages

Sensory attributes play an important role in determining the consumer acceptance of a new food product [35,49]. The photographs of test chicken sausages during 20 days of refrigerated storage (4 ± 1 °C) under aerobic packaging conditions did not show major visible discernible changes, except for color (Figure 7).

In fact, scores for the color and appearance, flavor, texture and tenderness, and juiciness of products indicate either freshness or the extent of spoilage during storage due to microbial action, lipid and protein degradation, moisture loss, etc. [81]. The sensory quality attributes, such as color (7.3–4.9), flavor (7.4–4.8), texture and tenderness (7.5–5.4), juiciness (7.5–5.4), and overall acceptability (7.4–4.9), for the control and treated chicken sausages during storage are tabulated in Table 7.

Significant differences (*p* ≤ 0.05) in color and appearance scores of C1 and T3B sausages were noticed from day 5 onwards, and in the case of the others, from day 10 onwards. Among the treatments, T3B showed lower color and appearance scores, as the sausages developed an atypical or slightly darker color after cooking due to higher concentrations of our formulation. In contrast, T2B and T1B obtained significantly higher color and appearance scores (*p* ≤ 0.05), reflecting the colorimetric properties of the phytochemical combinations (Figure 7). Flavor scores revealed that the treated sausages (T1B, T2B, T3B) obtained significantly higher scores (*p* ≤ 0.05) than the controls (C1, C2). Among the treatments, T2B had significantly higher flavor scores (*p* ≤ 0.05). Significant differences (*p* ≤ 0.05) in flavor scores of the test sausages were observed from day 10.

Significant differences (*p* ≤ 0.05) in texture and tenderness scores of the control and treated sausages were observed from day 10 onwards. These scores correlate with shear force values (Table 5); sausage samples with higher shear force values had better texture and tenderness scores. The treated sausages (T1B, T2B, T3B) had significantly higher juiciness scores (*p* ≤ 0.05) than the controls (C1, C2). During storage, mean juiciness scores decreased significantly (*p* ≤ 0.05) from day 10 onward for both treated and control (C1) samples.

The overall acceptability scores for C1 (7.1–4.9), C2 (7.4–5.9), T1B (7.3–6), T2B (7.4–6) and T3B (7.1–5.4) from day 0 and day 20 of refrigerated storage revealed significant (*p* ≤ 0.05) differences in the acceptance of control and treated sausages. Significant differences (*p* ≤ 0.05) in overall acceptability scores of the test sausages were markedly observed from day 5 and, for some (T2B and T3B) sausages, from day 10 onwards. The overall acceptability scores for T3B were significantly (*p* ≤ 0.05) lower than those of C2 and other treatments, but higher than those of C1. This is mainly due to the product’s atypical, darker color, which resulted in lower color and appearance scores. C1 obtained significantly lower scores (*p* ≤ 0.05) than other test sausages during storage. T2B and T1B obtained significantly (*p* ≤ 0.05) better overall acceptability scores than other sausages on day 20 of storage. The incorporation of our formulation did not adversely affect the acceptability of the chicken sausages, except at higher concentration (T3B), where an atypical color was seen.

These findings are corroborated by the report of Serdaroğlu et al. [74], who found no difference in the overall acceptability of heat-treated fermented sausages using arugula leaf extract and barberry extract as natural nitrite substitutes, either in combination or separately. While using radish powder (0.5 and 1.0%) and oregano essential oil (100 mg/kg) as nitrite substitutes in fermented cooked sausages, Ozaki et al. [34] also noticed equal or even higher sensory acceptance and consumer preference of treated sausages than the control.

## 4. Conclusions

The current study contributes significantly to the existing, but limited, scientific literature on natural nitrite replacements, both globally and in India. This study demonstrated that the novel phytochemical formulation, comprising roselle flower powder, moringa flower powder, and cinnamon essential oil in a ratio of 5.5:4:0.5, showed excellent antioxidant, broad-spectrum antimicrobial (including anticlostridial activity), and appealing colorimetric properties. This natural preservative retained its antimicrobial activity and showed excellent thermostability at varying temperatures (37 °C, 70 °C, and 100 °C) and acidic pH (2, 4, and 6), and remained stable for up to 30 days under refrigerated (4 ± 1 °C) conditions. Further, the formulation was non-cytotoxic in a hemolysis assay with sheep erythrocytes and an MTT assay using Vero cells and had no or negligible negative impact on gut lactobacilli. The incorporation of our formulation (0.25%, 0.5%, and 1%) in chicken sausages alone, or in combination with chemical nitrite, to replace nitrite resulted in an improvement in the quality and shelf life of treated sausages under aerobic packaging during refrigerated (4 ± 1 °C) storage for 20 days. Although complete nitrite replacement with natural preservatives significantly (*p* ≤ 0.05) improved the physicochemical and microbiological qualities of the treated sausages, sensory color was compromised by the development of an atypical, darker color. Therefore, a partial replacement of sodium nitrite (1/2–2/3rd, i.e., 75–100 ppm) by the natural preservative (formulation) described here could be considered for use.

Furthermore, this study highlights the need for future research to investigate the synergistic effects of combinations or blends of plant-derived ingredients as sustainable, green alternatives to sodium nitrite and other synthetic preservatives. Such approaches may facilitate the development of healthier processed meat products with enhanced quality, safety, and shelf life. Future efforts should also focus on validating their efficacy through comprehensive food safety assessments, formulation optimization, and large-scale production trials to support industrial application.

## Figures and Tables

**Figure 1:**
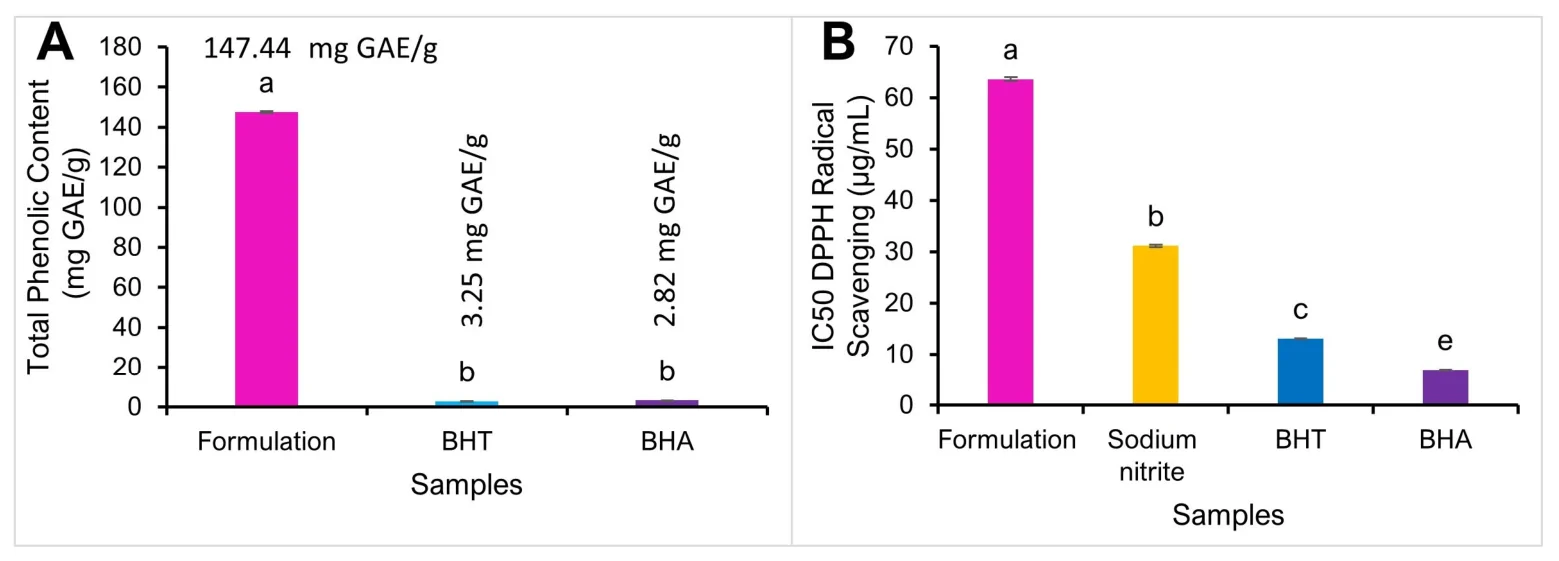
Total phenolic content (**A**) and DPPH radical-scavenging activity (**B**) of phytochemical formulation as compared to standard chemical antioxidants. BHT: butylated hydroxytoluene; BHA: butylated hydroxyanisole. Graphs showing different letters a, b, c, e are significantly different.

**Figure 2:**
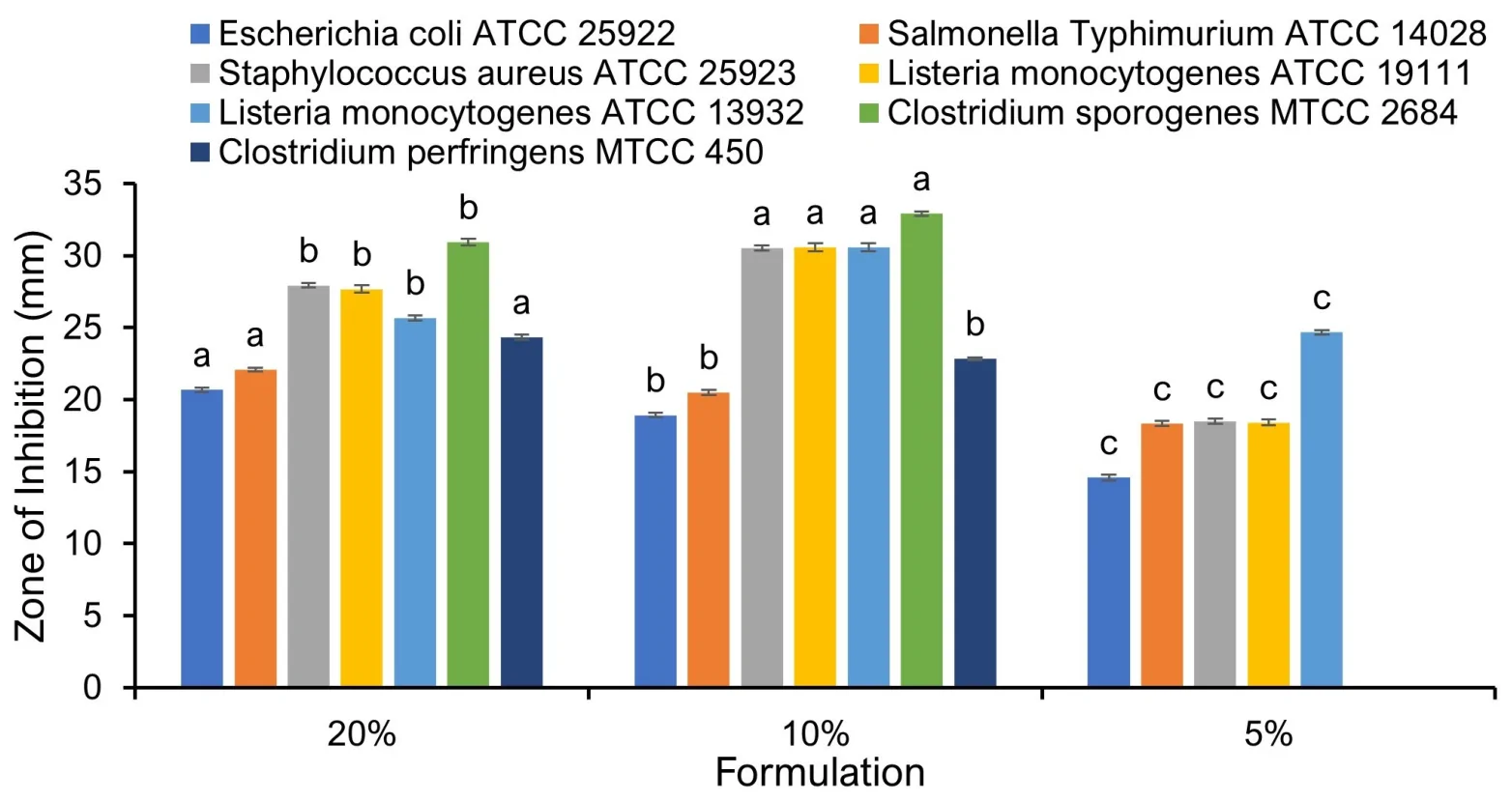
Antimicrobial activity (zone of inhibition) of phytochemical formulation against foodborne pathogens. Graphs showing different letters a, b, c are significantly different.

**Figure 3:**
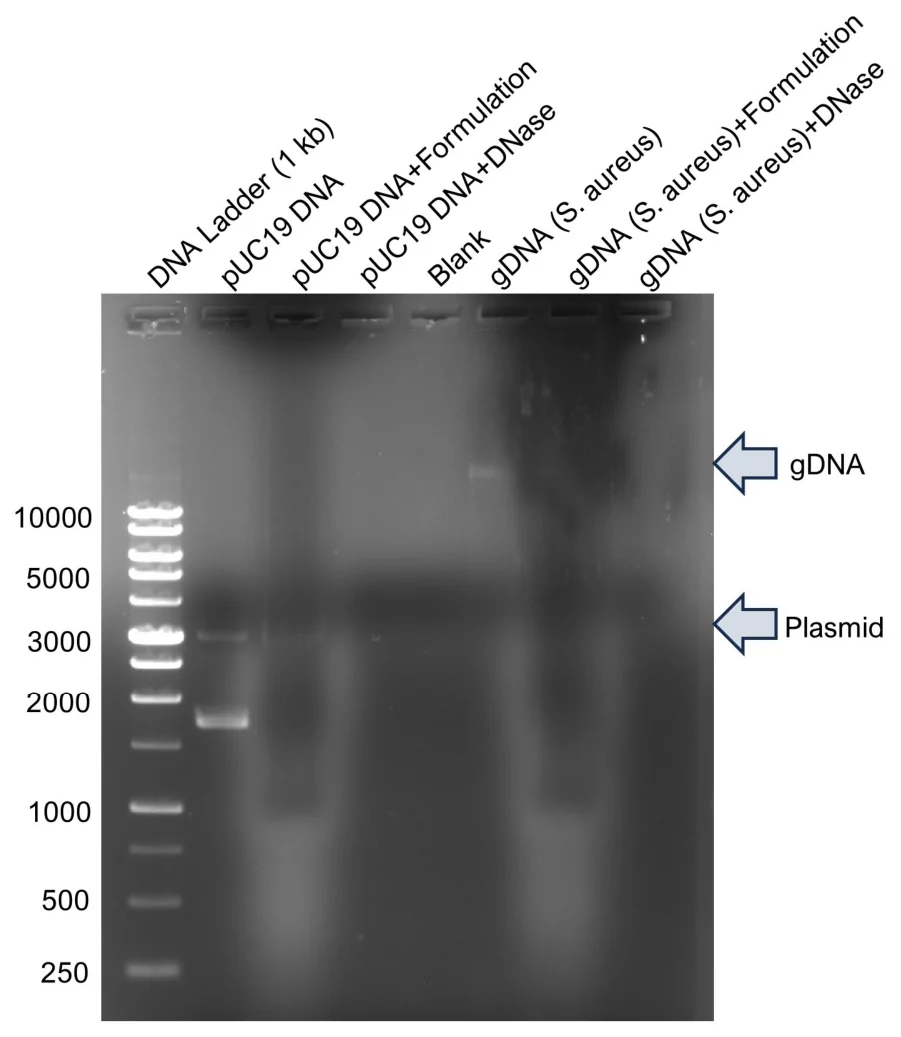
Gel image showing the extent of bacterial genomic DNA (gDNA) and plasmid DNA (pUC19) damage by the phytochemical formulation.

**Figure 4:**
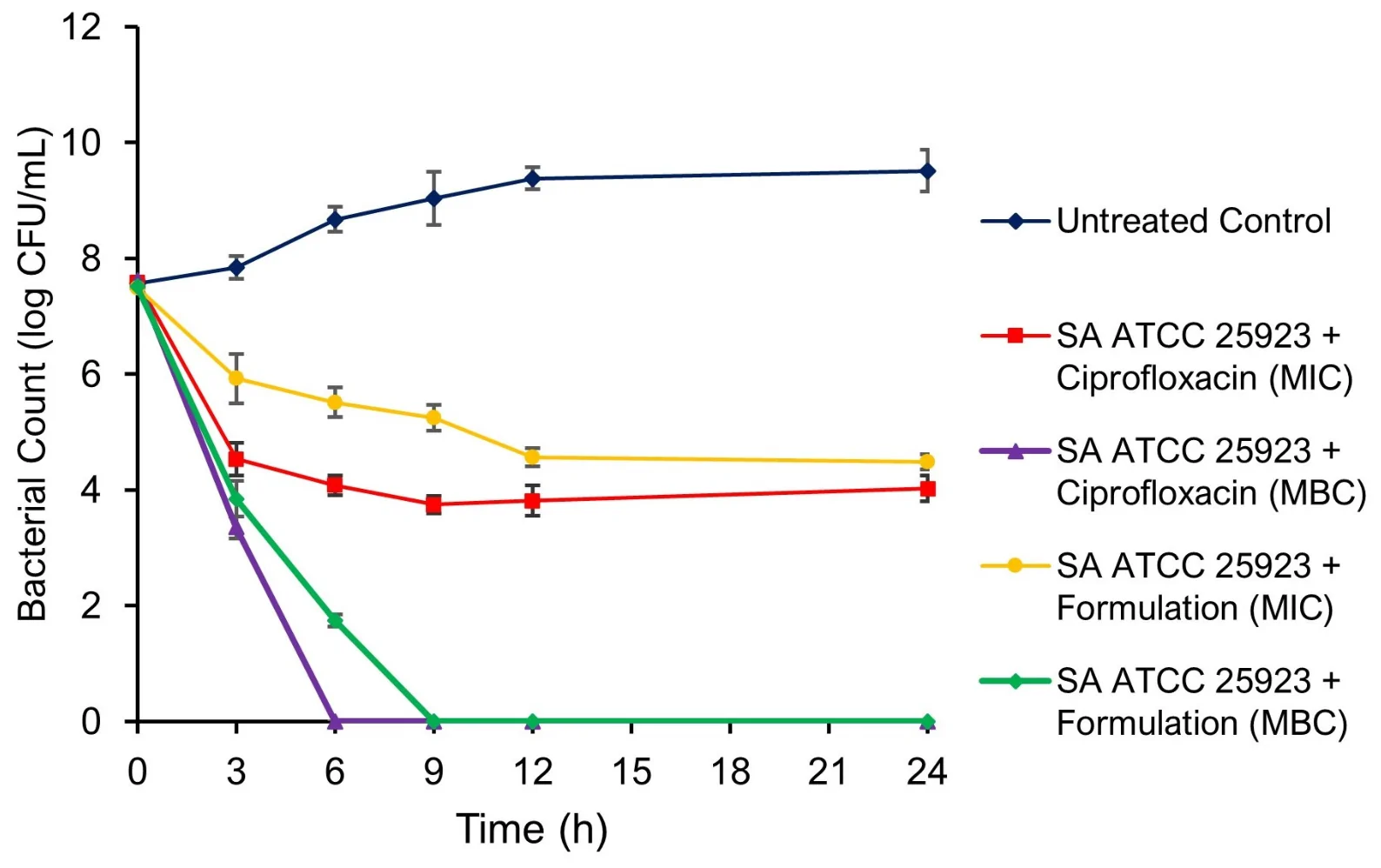
Time-kill assay showing dose- and time-dependent growth kinetics of *Staphylococcus aureus* (SA, ATCC 25923) using the phytochemical formulation.

**Figure 5:**
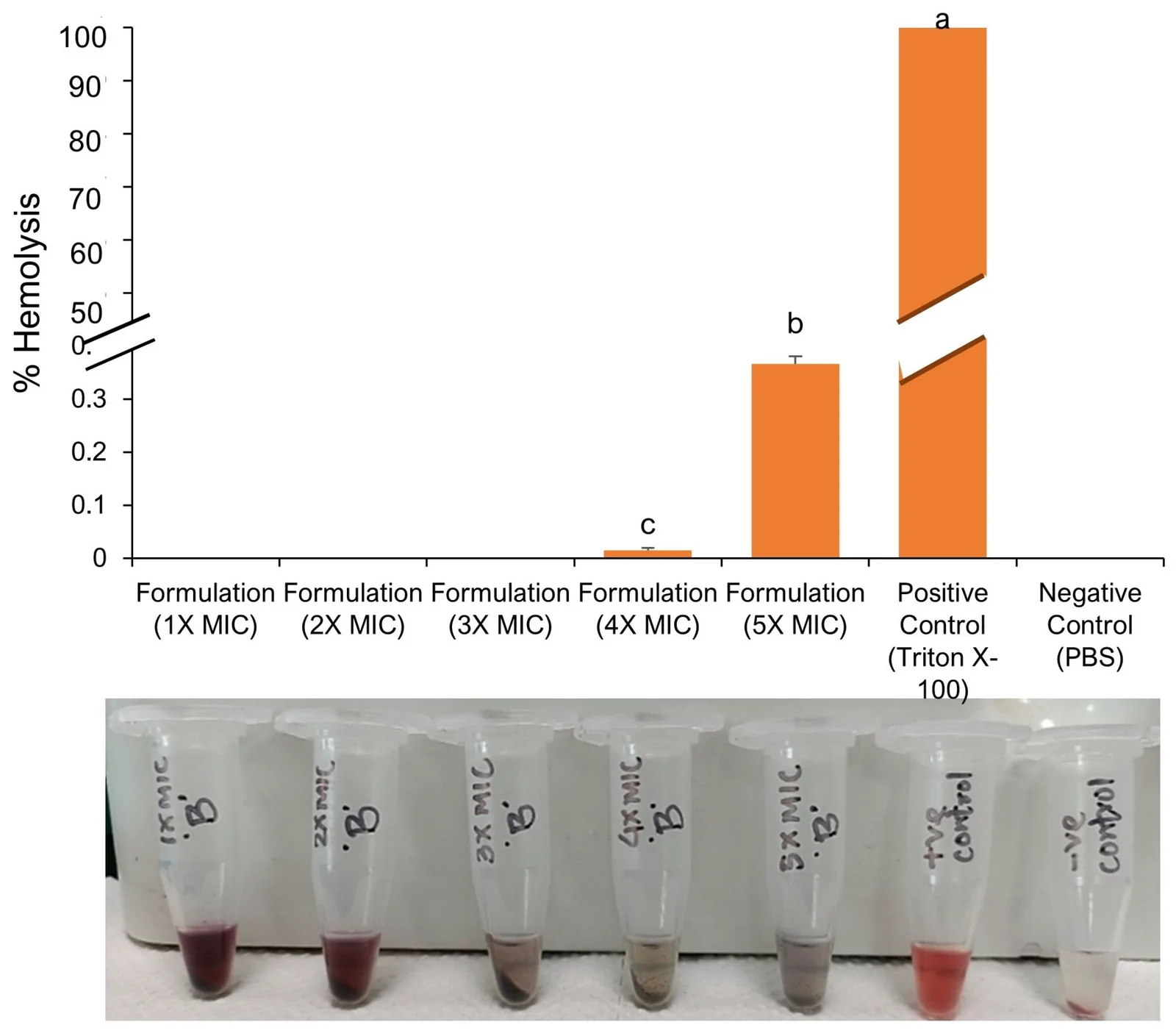
Hemolysis assay of phytochemical formulation using 5% sheep red blood cells. (**Top panel**) quantitative plot; (**Bottom panel**) corresponding photographs. Graphs showing different letters a, b, c are significantly different.

**Figure 6:**
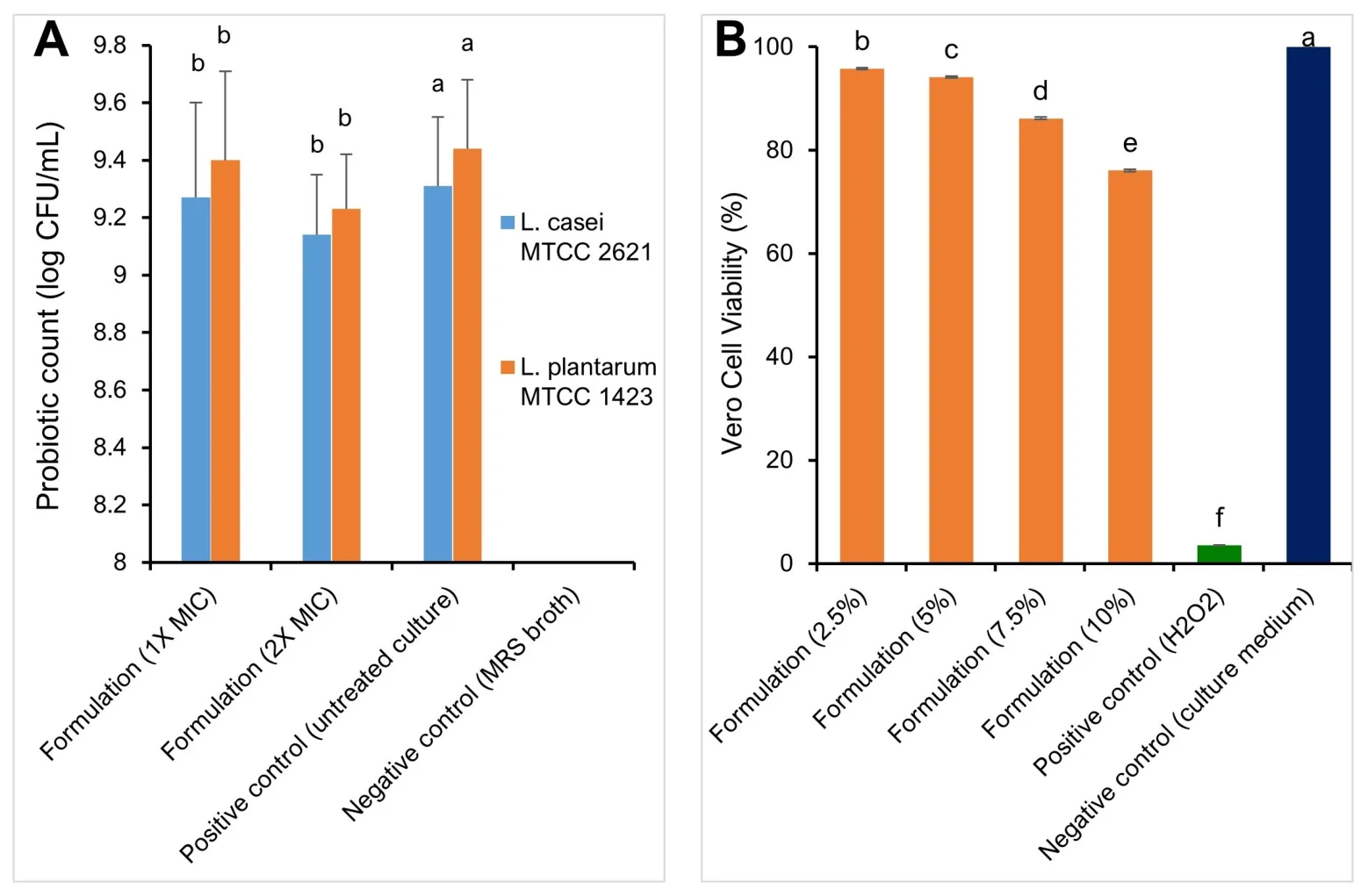
Effect of phytochemical formulation on the growth of probiotic lactobacilli (**A**) and Vero cell viability (**B**) at different concentrations of formulation in the 3-(4.5-dimethylthiazolyl-2)-2, 5-diphenyltetrazolium bromide (MTT) assay. Graphs showing different letters a, b, c, d, e, f are significantly different.

**Figure 7:**
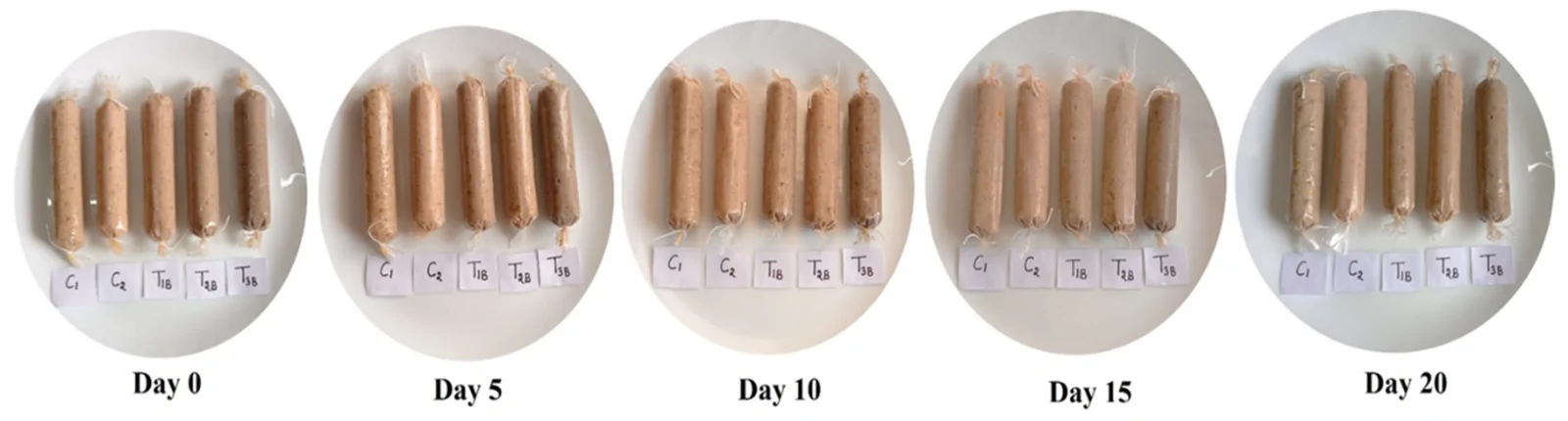
Test chicken sausages during 20 days of refrigerated storage (4 ± 1 °C) under aerobic packaging conditions. Sample labels are C1: no sodium nitrite or phytochemical formulation; C2: 150 ppm sodium nitrite + no phytochemical formulation; T1B (Treatment 1 Blend): 75 ppm sodium nitrite + 0.25% phytochemical formulation; T2B (Treatment 2 Blend): 50 ppm sodium nitrite + 0.5% phytochemical formulation; T3B (Treatment 3 Blend): no sodium nitrite + 1% phytochemical formulation.

**Table 1:** Total Extractable Components, pH, instrumental color, and residual nitrite content of phytochemical formulation.

Parameter	Phytochemical Formulation
TEC (%)	26.79 ± 0.23
pH	3.28 ± 0.03
Instrumental Color	
Lightness (L*)	21.87 ± 0.39
Redness (a*)	19.88 ± 0.41
Yellowness (b*)	13.43 ± 0.31
Hue angle (h_ab_)	6.20 ± 0.35
Chroma (C*_ab_)	25.01 ± 0.56
RN content	<D.C.

Values are average ± SEM. TEC: Total Extractable Components, RN: residual nitrite, <D.C.—below detection capability.

**Table 2:** Determination of MIC, MBC, and tolerance level of phytochemical formulation.

Bacteria	Phytochemical Formulation (RFP + MFP + CmEO)			Ciprofloxacin		
	MIC (mg/mL)	MBC (mg/mL)	Tolerance Level	MIC (mg/mL)	MBC (mg/mL)	Tolerance Level
EC 25922	0.390 ± 0.00 ^b^	0.781 ± 0.00 ^a^	2.00 ± 0.00	0.125 ± 0.00 ^b^	0.250 ± 0.00 ^b^	2.00 ± 0.00
ST 14028	0.195 ± 0.00 ^c^	0.195 ± 0.00 ^b^	1.00 ± 0.00	0.125 ± 0.00 ^b^	0.250 ± 0.00 ^b^	2.00 ± 0.00
SA 25923	0.781±0.00 ^a^	0.781 ± 0.00 ^a^	1.00 ± 0.00	0.250 ± 0.00 ^a^	0.500 ± 0.00 ^a^	2.00 ± 0.00
LM 19111	0.781±0.00 ^a^	0.781 ± 0.00 ^a^	1.00 ± 0.00	0.250 ± 0.00 ^a^	0.500 ± 0.00 ^a^	2.00 ± 0.00

Mean (n = 6) ± SE bearing different superscripts in a column differ significantly (*p* ≤ 0.05). RFP: roselle flower powder, MFP: moringa flower powder; CmEO: cinnamon essential oil, EC: *Escherichia coli*; ST: *Salmonella typhimurium*; SA: *Staphylococcus aureus*; LM: *Listeria monocytogenes*; MIC: minimum inhibitory concentration; MBC: minimum bactericidal concentration.

**Table 3:** Stability assays showing antimicrobial activity of the phytochemical formulation in terms of ZOI at varying temperatures, pH, and storage days.

Parameter	ZOI (mm)	
	*Salmonella typhimurium* ATCC 14028	*Staphylococcus aureus* ATCC 25923
Temperature		
37 °C	22.00 ± 0.26 ^a^	28.67 ± 0.17 ^a^
70 °C	20.00 ± 0.22 ^b^	27.00 ± 0.26 ^b^
100 °C	17.75 ± 0.17 ^c^	23.83 ± 0.25 ^c^
pH		
2	19.50 ± 0.18 ^c^	22.67 ± 0.25 ^c^
4	20.83 ± 0.21 ^b^	27.67 ± 0.21 ^b^
6	21.75 ± 0.25 ^a^	28.42 ± 0.15 ^a^
8	0.00 ± 0.00 ^d^	0.00 ± 0.00 ^d^
Storage days (4 ± 1 °C)		
0	22.00 ± 0.26 ^a^	28.67 ± 0.17 ^a^
15	21.25 ± 0.17 ^a^	28.33 ± 0.25 ^a^
30	20.00 ± 0.26 ^b^	27.25 ± 0.34 ^b^

Mean (n = 6) ± SE; bearing different superscripts in a column differ significantly (*p* ≤ 0.05). ZOI: zone of inhibition.

**Table 4:** Expressible water (%), proximate composition, and calorific value of control and chicken sausages treated with phytochemical formulation.

Treatments	Expressible Water (%)	Proximate Composition (%)					Calorific Value (kcal/100 g)
		Moisture (%)	Crude Protein (%)	Crude Fat (%)	Total Ash (%)	Carbohydrate (%)	
C1 (Negative control)	25.00 ± 0.29 ^a^	66.02 ± 0.12 ^d^	16.07 ± 0.33 ^a^	13.04 ± 0.19 ^a^	2.09 ± 0.03 ^c^	2.76 ± 0.03 ^c^	193.09 ± 0.16 ^a^
C2 (Positive control)	23.73 ± 0.13 ^b^	66.02 ± 0.35 ^d^	16.06 ± 0.16 ^a^	13.03 ± 0.25 ^a^	2.11 ± 0.09 ^c^	2.76 ± 0.05 ^c^	192.91 ± 0.14 ^a^
T1B	23.86 ± 0.20 ^b^	66.32 ± 0.36 ^c^	16.03 ± 0.14 ^a^	12.70 ± 0.18 ^b^	2.13 ± 0.09 ^c^	2.81 ± 0.05 ^bc^	190.04 ± 0.20 ^b^
T2B	22.78 ± 0.17 ^c^	66.73 ± 0.52 ^b^	15.76 ± 0.26 ^b^	12.34 ± 0.19 ^c^	2.23 ± 0.11 ^b^	2.93 ± 0.05 ^ab^	186.19 ± 0.15 ^c^
T3B	22.00 ± 0.13 ^c^	67.24 ± 0.31 ^a^	15.30 ± 0.32 ^b^	12.14 ± 0.20 ^d^	2.32 ± 0.10 ^a^	2.98 ± 0.05 ^a^	182.72 ± 0.11 ^d^

Mean (n = 6) ± SE bearing different superscripts in a column differ significantly (*p* ≤ 0.05). C1: no sodium nitrite or phytochemical formulation (negative control); C2: 150 ppm sodium nitrite + no phytochemical formulation (positive control), T1B (Treatment 1 Blend): 75 ppm sodium nitrite + 0.25% phytochemical formulation; T2B (Treatment 2 Blend): 50 ppm sodium nitrite + 0.5% phytochemical formulation; T3B (Treatment 3 Blend): no sodium nitrite + 1% phytochemical formulation.

**Table 5:** Physicochemical quality changes in control and chicken sausages treated with phytochemical formulation during storage.

Treatment	Day 0	Day 5	Day 10	Day 15	Day 20
TBARS value (mg MDA/kg)					
C1	0.287 ± 0.008 ^D^	0.321 ± 0.006 ^D^	0.510 ± 0.017 ^aC^	1.019 ± 0.039 ^aB^	2.031 ± 0.039 ^aA^
C2	0.286 ± 0.009 ^D^	0.313 ± 0.009 ^D^	0.402 ± 0.023 ^bC^	0.593 ± 0.009 ^bB^	1.051 ± 0.025 ^bA^
T1B	0.278 ± 0.008 ^D^	0.300 ± 0.005 ^D^	0.391 ± 0.022 ^bC^	0.498 ± 0.004 ^cB^	0.941 ± 0.014 ^cA^
T2B	0.281 ± 0.009 ^D^	0.303 ± 0.009 ^D^	0.351 ± 0.016 ^bC^	0.495 ± 0.012 ^cB^	0.773 ± 0.018 ^dA^
T3B	0.280 ± 0.008 ^D^	0.301 ± 0.009 ^D^	0.343 ± 0.012 ^bC^	0.484 ± 0.010 ^cB^	0.761 ± 0.019 ^dA^
RN content (mg/kg)					
C1	<D.C.	<D.C.	<D.C.	<D.C.	<D.C.
C2	80.35 ± 0.19 ^aA^	70.67 ± 0.35 ^aB^	62.61 ± 0.13 ^aC^	55.44 ± 0.56 ^aC^	46.95 ± 0.18 ^aD^
T1B	47.14 ± 0.42 ^bA^	42.18 ± 0.31 ^bB^	34.12 ± 0.40 ^bC^	29.68 ± 0.51 ^bD^	26.46 ± 0.19 ^bD^
T2B	31.10 ± 0.36 ^cA^	27.64 ± 0.24 ^cA^	22.19 ± 0.34 ^cB^	20.75 ± 0.50 ^cB^	16.03 ± 0.52 ^cC^
T3B	<D.C.	<D.C.	<D.C.	<D.C.	<D.C.
Shear force (kgf/cm^2^)					
C1	1.88 ± 0.008 ^aA^	1.85 ± 0.008 ^aA^	1.76 ± 0.010 ^bB^	1.66 ± 0.007 ^bC^	1.44 ± 0.008 ^cD^
C2	1.85 ± 0.009 ^bA^	1.84 ± 0.005 ^aA^	1.80 ± 0.010 ^aB^	1.66 ± 0.016 ^bC^	1.58 ± 0.019 ^bD^
T1B	1.84 ± 0.008 ^bA^	1.82 ± 0.006 ^bAB^	1.79 ± 0.016 ^abB^	1.68 ± 0.012 ^bC^	1.59 ± 0.013 ^bD^
T2B	1.81 ± 0.007 ^cA^	1.80 ± 0.005 ^bcA^	1.76 ± 0.009 ^bB^	1.72 ± 0.005 ^aC^	1.64 ± 0.013 ^aD^
T3B	1.80 ± 0.010 ^cA^	1.79 ± 0.005 ^cA^	1.78 ± 0.007 ^abA^	1.73 ± 0.010 ^aB^	1.64 ± 0.013 ^aC^
Cured pigments (mg/kg)					
C1	16.41 ± 0.04 ^eA^	15.93 ± 0.04 ^dB^	13.69 ± 0.12 ^dC^	10.53 ± 0.13 ^dD^	5.83 ± 0.02 ^dE^
C2	57.63 ± 0.03 ^aA^	57.76 ± 0.16 ^aA^	56.40 ± 0.19 ^aB^	53.88 ± 0.18 ^aC^	49.34 ± 0.18 ^aD^
T1B	50.30 ± 0.02 ^bB^	51.49 ± 0.03 ^bA^	50.15 ± 0.03 ^bC^	47.24 ± 0.03 ^bD^	42.35 ± 0.04 ^bE^
T2B	40.78 ± 0.09 ^cA^	41.24 ± 0.12 ^cA^	40.08 ± 0.12 ^cB^	37.29 ± 0.14 ^cC^	31.22 ± 0.11 ^cD^
T3B	16.62 ± 0.04 ^dA^	15.69 ± 0.05 ^dB^	13.81 ± 0.05 ^dC^	10.79 ± 0.03 ^dD^	5.59 ± 0.01 ^dE^
Total pigments (mg/kg)					
C1	65.84 ± 0.04 ^eA^	63.54 ± 0.10 ^eB^	60.77 ± 0.10 ^eC^	56.49 ± 0.16 ^eD^	50.25 ± 0.25 ^eE^
C2	83.02 ± 0.17 ^dA^	83.39 ± 0.15 ^dA^	80.87 ± 0.11 ^dB^	77.69 ± 0.08 ^dC^	71.36 ± 0.04 ^dD^
T1B	85.31 ± 0.14 ^cA^	85.01 ± 0.03 ^cA^	83.93 ± 0.06 ^cB^	81.00 ± 0.08 ^cC^	75.69 ± 0.21 ^cD^
T2B	90.09 ± 0.18 ^bB^	91.40 ± 0.15 ^bA^	90.32 ± 0.15 ^bB^	87.43 ± 0.29 ^bC^	80.82 ± 0.14 ^bD^
T3B	95.26 ± 0.24 ^aA^	94.19 ± 0.19 ^aB^	92.30 ± 0.18 ^aC^	89.13 ± 0.20 ^aD^	84.03 ± 0.09 ^aE^
Curing efficiency (%)					
C1	24.93 ± 0.06 ^dA^	25.07 ± 0.03 ^dA^	22.52 ± 0.16 ^dB^	18.64 ± 0.19 ^dC^	11.61 ± 0.05 ^dD^
C2	69.42 ± 0.16 ^a^	69.27 ± 0.29 ^a^	69.73 ± 0.22 ^a^	69.35 ± 0.19 ^a^	69.15 ± 0.26 ^a^
T1B	58.96 ± 0.10 ^bC^	60.57 ± 0.04 ^bA^	59.76 ± 0.03 ^bB^	58.33 ± 0.05 ^bD^	55.95 ± 0.12 ^bE^
T2B	45.27 ± 0.04 ^cA^	45.12 ± 0.06 ^cA^	44.38 ± 0.06 ^cB^	42.65 ± 0.06 ^cC^	38.62 ± 0.07 ^cD^
T3B	17.45 ± 0.04 ^eA^	16.66 ± 0.05 ^eB^	14.96 ± 0.04 ^eC^	12.11 ± 0.02 ^eD^	6.65 ± 0.01 ^eE^

Mean (n = 6) ± SE bearing different lower-case superscripts (a, b, c, d, e) in a column and upper-case superscripts (A, B, C, D, E) in a row differ significantly (*p* ≤ 0.05). <D.C.—Below Detection Capability. C1: no sodium nitrite or phytochemical formulation (negative control); C2: 150 ppm sodium nitrite + no phytochemical formulation (positive control), T1B (Treatment 1 Blend): 75 ppm sodium nitrite + 0.25% phytochemical formulation; T2B (Treatment 2 Blend): 50 ppm sodium nitrite + 0.5% phytochemical formulation; T3B (Treatment 3 Blend): no sodium nitrite + 1% phytochemical formulation.

**Table 6:** Microbiological quality changes in control and chicken sausages treated with phytochemical formulation during storage.

Total Viable Counts (log CFU/g)					
Treatment	Day 0	Day 5	Day 10	Day 15	Day 20
C1	1.58 ± 0.10 ^aE^	1.98 ± 0.22 ^aD^	2.77 ± 0.27 ^aC^	3.55 ± 0.31 ^aB^	4.65 ± 0.32 ^aA^
C2	1.41 ± 0.19 ^bE^	1.79 ± 0.20 ^bD^	2.45 ± 0.23 ^bC^	3.22 ± 0.24 ^bB^	4.13 ± 0.25 ^bA^
T1B	1.38 ± 0.09 ^bE^	1.71 ± 0.12 ^cD^	2.23 ± 0.21 ^cC^	3.08 ± 0.19 ^cB^	3.81 ± 0.27 ^cA^
T2B	1.30 ± 0.13 ^cE^	1.50 ± 0.15 ^dD^	2.19 ± 0.20 ^cC^	2.88 ± 0.21 ^dB^	3.69 ± 0.16 ^dA^
T3B	1.28 ± 0.09 ^cE^	1.47 ± 0.08 ^dD^	2.04 ± 0.15 ^dC^	2.69 ± 0.10 ^eB^	3.52 ± 0.23 ^eA^
Psychrophile Counts (log CFU/g)					
C1	ND	ND	1.21 ± 0.03 ^aC^	2.38 ± 0.08 ^aB^	3.56 ± 0.07 ^aA^
C2	ND	ND	ND	1.29 ± 0.19 ^bB^	2.41 ± 0.14 ^bA^
T1B	ND	ND	ND	1.17 ± 0.21 ^bB^	1.94 ± 0.23 ^cA^
T2B	ND	ND	ND	ND	1.45 ± 0.21 ^dA^
T3B	ND	ND	ND	ND	1.24 ± 0.17 ^eA^
*Staphylococcus aureus* Counts (log CFU/g)					
C1	ND	ND	1.30 ± 0.14 ^aC^	2.19 ± 0.23 ^bB^	2.55 ± 0.24 ^aA^
C2	ND	ND	ND	1.19 ± 0.16 ^bB^	2.00 ± 0.28 ^bA^
T1B	ND	ND	ND	1.04 ± 0.05 ^cB^	1.78 ± 0.11 ^cA^
T2B	ND	ND	ND	ND	1.51 ± 0.10 ^dA^
T3B	ND	ND	ND	ND	1.34 ± 0.12 ^eA^

Mean (n = 6) ± SE marked with different superscripts (a, b, c, d, e) in a column and (A, B, C, D, E) in a row differ significantly (*p* ≤ 0.05). N.D.—Not Detected. C1: no sodium nitrite or phytochemical formulation (negative control); C2: 150 ppm nitrite + no phytochemical formulation (positive control), T1B (Treatment 1 Blend): 75 ppm sodium nitrite + 0.25% phytochemical formulation; T2B (Treatment 2 Blend): 50 ppm sodium nitrite + 0.5% phytochemical formulation; T3B (Treatment 3 Blend): no sodium nitrite + 1% phytochemical formulation.

**Table 7:** Sensory quality changes in control and chicken sausages treated with phytochemical formulation during storage.

Color and Appearance					
Treatment	Day 0	Day 5	Day 10	Day 15	Day 20
C1	7.0 ± 0.22 ^bA^	6.9 ± 0.20 ^bB^	6.1 ± 0.12 ^bC^	5.6 ± 0.10 ^dD^	4.9 ± 0.28 ^dE^
C2	7.3 ± 0.16 ^aA^	7.3 ± 0.27 ^aA^	7.1 ± 0.21 ^aB^	6.7 ± 0.34 ^bC^	5.9 ± 0.20 ^bD^
T1B	7.3 ± 0.14 ^aA^	7.3 ± 0.21 ^aA^	7.0 ± 0.12 ^aB^	6.8 ± 0.23 ^aC^	6.3 ± 0.23 ^aD^
T2B	7.3 ± 0.17 ^aA^	7.4 ± 0.25 ^aA^	7.1 ± 0.15 ^aB^	6.8 ± 0.16 ^aC^	6.3 ± 0.16 ^aD^
T3B	6.7 ± 0.30 ^cA^	6.6 ± 0.23 ^cB^	6.2 ± 0.16 ^bC^	5.8 ± 0.19 ^cD^	5.2 ± 0.23 ^cE^
Flavor					
C1	7.1 ± 0.14 ^cA^	7.1 ± 0.10 ^cA^	6.6 ± 0.10 ^cB^	5.8 ± 0.17 ^cC^	4.8 ± 0.27 ^cD^
C2	7.3 ± 0.13 ^abA^	7.3 ± 0.13 ^aA^	6.9 ± 0.12 ^aB^	6.4 ± 0.20 ^aC^	5.7 ± 0.27 ^aD^
T1B	7.3 ± 0.18 ^abA^	7.3 ± 0.12 ^aA^	6.9 ± 0.19 ^aB^	6.3 ± 0.12 ^bC^	5.7 ± 0.20 ^aD^
T2B	7.4 ± 0.20 ^aA^	7.3 ± 0.27 ^aA^	6.9 ± 0.27 ^aB^	6.4 ± 0.21 ^aC^	5.8 ± 0.21 ^aD^
T3B	7.3 ± 0.17 ^bA^	7.2 ± 0.20 ^bA^	6.8 ± 0.20 ^bB^	6.3 ± 0.21 ^bC^	5.6 ± 0.23 ^bD^
Texture and Tenderness					
C1	7.3 ± 0.09 ^cA^	7.2 ± 0.09 ^cB^	6.8 ± 0.14 ^cC^	6.3 ± 0.21 ^dD^	5.4 ± 0.29 ^cE^
C2	7.5 ± 0.09 ^aA^	7.4 ± 0.10 ^bB^	7.0 ± 0.10 ^bC^	6.4 ± 0.10 ^cD^	5.8 ± 0.16 ^bE^
T1B	7.5 ± 0.10 ^aA^	7.4 ± 0.12 ^aA^	7.1 ± 0.18 ^aB^	6.6 ± 0.12 ^aC^	6.0 ± 0.12 ^aD^
T2B	7.5 ± 0.15 ^aA^	7.5 ± 0.22 ^aA^	7.1 ± 0.21 ^aB^	6.7 ± 0.23 ^aC^	6.0 ± 0.22 ^aD^
T3B	7.4 ± 0.12 ^bA^	7.4 ± 0.19 ^bA^	7.0 ± 0.26 ^bB^	6.5 ± 0.22 ^bC^	5.8 ± 0.24 ^bD^
Juiciness					
C1	7.3 ± 0.14 ^bA^	7.2 ± 0.14 ^cB^	6.8 ± 0.21 ^cC^	6.3 ± 0.28 ^cD^	5.4 ± 0.34 ^cE^
C2	7.3 ± 0.18 ^bA^	7.3 ± 0.18 ^bA^	7.1 ± 0.22 ^bB^	6.7 ± 0.22 ^bC^	6.0 ± 0.20 ^bE^
T1B	7.5 ± 0.09 ^aA^	7.4 ± 0.14 ^bB^	7.1 ± 0.14 ^bC^	6.7 ± 0.17 ^bD^	6.1 ± 0.17 ^bE^
T2B	7.5 ± 0.14 ^aA^	7.5 ± 0.14 ^aA^	7.1 ± 0.13 ^bB^	6.7 ± 0.20 ^bC^	6.1 ± 0.27 ^bD^
T3B	7.5 ± 0.11 ^aA^	7.5 ± 0.16 ^aA^	7.2 ± 0.16 ^aB^	6.8 ± 0.16 ^aC^	6.2 ± 0.14 ^aD^
Overall acceptability					
C1	7.1 ± 0.15 ^cA^	7.0 ± 0.21 ^cA^	6.7 ± 0.31 ^cB^	5.9 ± 0.42 ^dC^	4.9 ± 0.42 ^dD^
C2	7.4 ± 0.15 ^aA^	7.2 ± 0.21 ^bB^	7.0 ± 0.25 ^bC^	6.6 ± 0.32 ^aD^	5.9 ± 0.27 ^bE^
T1B	7.3 ± 0.19 ^bA^	7.3 ± 0.24 ^bA^	7.0 ± 0.24 ^bB^	6.5 ± 0.18 ^bC^	6.0 ± 0.14 ^aD^
T2B	7.4 ± 0.12 ^aA^	7.4 ± 0.15 ^aA^	7.1 ± 0.20 ^aB^	6.6 ± 0.16 ^aC^	6.0 ± 0.23 ^aD^
T3B	7.1 ± 0.15 ^cA^	7.0 ± 0.15 ^cB^	6.7 ± 0.16 ^cC^	6.1 ± 0.16 ^cD^	5.4 ± 0.13 ^cE^

Mean (n = 6) ± SE; bearing different lower-case superscripts (a, b, c, d) in a column and upper-case superscripts (A, B, C, D, E) in a row differ significantly (*p* ≤ 0.05). C1: no sodium nitrite or phytochemical formulation; C2: 150 ppm sodium nitrite + no phytochemical formulation; T1B (Treatment 1 Blend): 75 ppm sodium nitrite + 0.25% phytochemical formulation; T2B (Treatment 2 Blend): 50 ppm sodium nitrite + 0.5% phytochemical formulation; T3B (Treatment 3 Blend): no sodium nitrite + 1% phytochemical formulation.

## Data Availability

All data are presented in the manuscript as primary or Supplementary Materials.

## Supplementary Materials

The following supporting information can be downloaded at https://www.mdpi.com/article/10.3390/foods15193450/s1, Figure S1: Identification of phytochemical compounds in formulation using gas chromatography–mass spectrometry (GC–MS); Table S1: Antimicrobial activity in terms of zone of inhibition (mm) for standardization and development of phytochemical formulation; Table S2: Identification of phytochemical compounds in the formulation using gas chromatography–mass spectrometry (GC–MS), related to Figure S1.
