# Supplementary material for: An Innovative Blend of Cinnamon Essential Oil, Roselle, and Moringa Flower Powders as a Natural Alternative to Sodium Nitrite in Chicken Sausage Processing
Source: Foods. 2026 Sep 27;15(19):3450. doi: 10.3390/foods15193450 (PMC13664000; doi:10.3390/foods15193450)
Supplement: Supplementary file 1 [file foods-15-03450-s001.zip › foods-4531919-supplementary.pdf]

Supplementary Data

An Innovative Blend of Cinnamon Essential Oil, Roselle, and Moringa Flower Powders as a Natural Alternative to Nitrite in Chicken Sausage Processing

Annada Das[1,2], Subhasish Biswas[1], Kaushik Satyaprakash[3], Dipanwita Bhattacharya[3], Pramod K. Nanda[4], Gopal Patra[1,5], Arun K. Bhunia[5,6,7]* and Arun K. Das[4]*

Table S1. Antimicrobial activity in terms of zone of inhibition (mm) for standardization and development of phytochemical formulation.

Phytochemical Formulation (RFP + MFP + CmEO)	Ratio (RFP: MFP: CmEO) of plant components	EC 25922	ST 14028	SA 25923	LM 19111	LM 13932
1	4:4:2	22.00 ± 0.13^{a}	21.00 ± 0.13^{b}	28.67 ± 0.17^{b}	30.17 ± 0.31^{a}	26.00 ± 0.29^{b}
2	5.5:4:0.5	22.17 ± 0.11^{a}	22.00 ± 0.26^{a}	30.00 ± 0.18^{a}	30.67 ± 0.28^{a}	26.00 ± 0.13^{b}
3	5:4:1	17.00 ± 0.29^{b}	14.67 ± 0.25^{c}	22.75 ± 0.28^{c}	24.00 ± 0.13^{b}	28.00 ± 0.13^{a}

*Mean (n=6) ± SE bearing different superscripts in a column differ significantly ($p < 0.05$).

RFP: roselle flower powder, MFP: moringa flower powder; CmEO: cinnamon essential oil, EC: *Escherichia coli;* ST: *Salmonella* Typhimurium; SA: *Staphylococcus aureus;* LM: *Listeria monocytogenes*

Table S2. Identification of phytochemical compounds in the formulation using Gas chromatography–mass spectrometry (GC-MS), related to Fig S1.

**Compound name**	**Common/ Trivial name**	**Formula**	**Mol. Wt. (g/mol)**	**Retention Time (RT) (min)**	**Area %**
Ethanone,1-(2,2-Dimethylcyclopentyl)-	Cyclopentyl methyl ketone derivative	$C_9H_{16}O$	140	7.37	0.121
2-Heptanol, 5-Ethyl-	Ethyl-substituted heptanol	$C_9H_{20}O$	144	28.53	0.228
6-Methyl-2-Heptanol, Trifluoroacetate	Trifluoroacetate ester of methyl heptanol	$C_{10}H_{17}F_3O_2$	226	29.15	0.152
(1s,15s)-Bicyclo[13.1.0] Hexadecan-2-One	(-)-Bourgeonal	$C_{16}H_{28}O$	236	31.27	0.157
6-Methyl-2-Heptanol, Heptafluorobutyrate	Heptafluorobutyrate ester of methyl heptanol	$C_{12}H_{17}F_7O_2$	326	31.51	0.154
Formic Acid, Oct-2-Yl Ester	Octyl formate	$C_9H_{18}O_2$	158	31.92	0.258
N-Cyclohexyl-N'-4-[N-Aziridyl] Butylurea	Substituted urea derivative	$C_{13}H_{25}N_3O$	239	32.08	0.248
6-Bromo-2,2-Dimethylcyclohexanone	Bromoacetone	$C_{18}H_{13}BrO$	204	33.16	0.415
5-Undecene, 8-Methyl-, (E)-	Macrocyclic ketone	$C_{12}H_{24}$	168	34.01	0.201
Pentadecafluorooctanoic Acid, Nonyl Ester	Flourinated fatty ester	$C_{17}H_{19}F_{15}O$	540	34.19	0.135
1,3,5-Tris (Cyclohexyl)Pent-1-Ene	Tricyclohexyl alkene	$C_{23}H_{40}$	316	5.1	0.474
1,12-Bis(2-Nitrophenoxy) Dodecane	Dinitrophenyl ether derivative	$C_{24}H_{32}N_2O_6$	444	5.32	0.351
Cyclohexane, 1,1'-(2-Propyl-1,3-Propanediyl) Bis-	Dicyclohexyl propane derivative	$C_{18}H_{34}$	250	5.45	0.604
Bicyclo [3.1.1] Heptan-2-One, 6,6-Dimethyl-	Nopinone / Pinocamphone	$C_9H_{14}O$	138	7.1	0.225
2,5-Furandione, 3-Dodecyl-	Dodecyl maleic anhydride	$C_{16}H_{26}O_3$	266	7.57	0.367
Cyclopentane, 2-(1-Hydroxy-2-Propyl)-1,3-Dimethyl-	Terpenoid alcohol derivative	$C_{10}H_{20}O$	156	8.03	0.146
Phosphonic Acid, 7-Octenyl-, Dibutyl Ester	Dibutyl octenyl phosphonate	$C_{16}H_{33}O_3P$	304	10.21	0.516
[1,1'-Bicyclohexyl]-4-Carboxylic Acid, 4'-Propyl-, 4-Fluorophenyl Ester	Aromatic ester derivative	$C_{22}H_{31}FO_2$	346	11.47	0.138
1,3,5-Tris(Cyclohexyl)Hex-1-Ene	Tricyclohexyl alkene	$C_{24}H_{42}$	330	13.09	0.236
Cyclohexane, 1,1'-(2-Ethyl-1,3-Propanediyl) Bis-	Dicyclohexyl ethylpropane	$C_{17}H_{32}$	236	15.31	0.289
1-Octanol, 3,7-Dimethyl-	Tetrahydrogeraniol/ (R)-Digydrocitronellol / Citronellol	$C_{10}H_{18}O$	158	7.37	0.824
Cyclohexanone, 4-Ethyl-	Ethyl cyclohexanone	$C_8H_{14}O$	126	7.38	0.129
1-Tetradecanol, 14-Chloro-	Chlorotetradecanol	$C_{14}H_{29}ClO$	248	8.46	0.24
2,10-Dodecadien-1-Ol, 3,7,11-Trimethyl-, (E)-(.+/-.)-	E-nerolidol	$C_{15}H_{28}O$	224	9.06	0.193
Oxiranecarboxaldehyde, 3-Methyl-3-(4-Methyl-3-Pentenyl)-	2,3-Epoxy-geranial (epoxy aldehyde)	$C_{10}H_{16}O_2$	168	13.06	0.146
6-Methyl-Cyclohex-2-En-1-Ol	Tetrahydro-o-kresol / Carveol (isomer)	$C_7H_{12}O$	112	14.78	0.666
4-Ethyl-1-Hexyn-3-Ol	Acetylenic alcohol	$C_8H_{14}O$	126	15.18	0.115

Cyclohexane, 1,2-Dimethyl-, Cis-	Cis-dimethyl cyclohexane	C_8H_{16}	112	16.23	0.82
Formic Acid, Decyl Ester	Decyl formate	$C_{11}H_{22}O_2$	186	16.25	0.351
Cyclodecane	Cyclodecane	$C_{10}H_{20}$	140	16.7	0.153

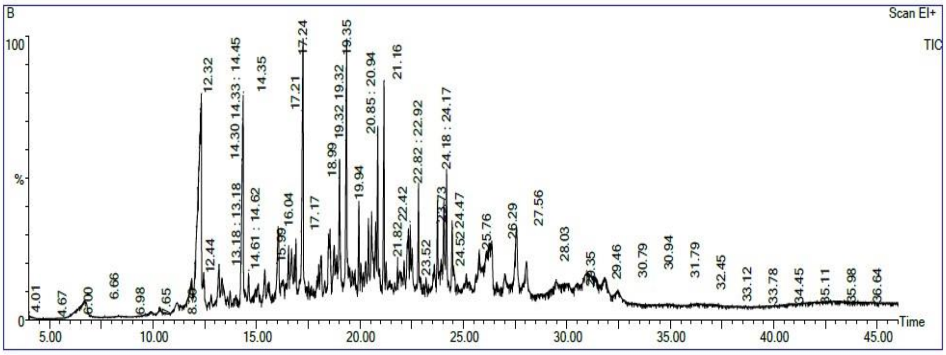

Figure S1: Identification of phytochemical compounds in formulation using Gas chromatography–mass spectrometry (GC-MS).
